# Positive end-expiratory pressure in COVID-19 acute respiratory distress syndrome: the heterogeneous effects

**DOI:** 10.1186/s13054-021-03839-4

**Published:** 2021-12-16

**Authors:** Davide Chiumello, Matteo Bonifazi, Tommaso Pozzi, Paolo Formenti, Giuseppe Francesco Sferrazza Papa, Gabriele Zuanetti, Silvia Coppola

**Affiliations:** 1 Department of Anesthesia and Intensive Care, ASST Santi Paolo e Carlo, San Paolo University Hospital, Via Di Rudini 9, Milan, Italy; 2 Department of Health Sciences, University of Milan, Milan, Italy; 3 Coordinated Research Center on Respiratory Failure, University of Milan, Milan, Italy; 4 Dipartimento di Scienze Neuroriabilitative, Casa di Cura del Policlinico, Milan, Italy

**Keywords:** SARS-CoV-2, COVID-19, Respiratory distress syndrome, Respiratory mechanics, Ventilator-induced lung injury

## Abstract

**Background:**

We hypothesized that as CARDS may present different pathophysiological features than classic ARDS, the application of high levels of end-expiratory pressure is questionable. Our first aim was to investigate the effects of 5–15 cmH_2_O of PEEP on partitioned respiratory mechanics, gas exchange and dead space; secondly, we investigated whether respiratory system compliance and severity of hypoxemia could affect the response to PEEP on partitioned respiratory mechanics, gas exchange and dead space, dividing the population according to the median value of respiratory system compliance and oxygenation. Thirdly, we explored the effects of an additional PEEP selected according to the Empirical PEEP-FiO_2_ table of the EPVent-2 study on partitioned respiratory mechanics and gas exchange in a subgroup of patients.

**Methods:**

Sixty-one paralyzed mechanically ventilated patients with a confirmed diagnosis of SARS-CoV-2 were enrolled (age 60 [54–67] years, PaO_2_/FiO_2_ 113 [79–158] mmHg and PEEP 10 [10–10] cmH_2_O). Keeping constant tidal volume, respiratory rate and oxygen fraction, two PEEP levels (5 and 15 cmH_2_O) were selected. In a subgroup of patients an additional PEEP level was applied according to an Empirical PEEP-FiO_2_ table (empirical PEEP). At each PEEP level gas exchange, partitioned lung mechanics and hemodynamic were collected.

**Results:**

At 15 cmH_2_O of PEEP the lung elastance, lung stress and mechanical power were higher compared to 5 cmH_2_O. The PaO_2_/FiO_2_, arterial carbon dioxide and ventilatory ratio increased at 15 cmH_2_O of PEEP. The arterial–venous oxygen difference and central venous saturation were higher at 15 cmH_2_O of PEEP. Both the mechanics and gas exchange variables significantly increased although with high heterogeneity. By increasing the PEEP from 5 to 15 cmH_2_O, the changes in partitioned respiratory mechanics and mechanical power were not related to hypoxemia or respiratory compliance. The empirical PEEP was 18 ± 1 cmH_2_O. The empirical PEEP significantly increased the PaO_2_/FiO_2_ but also driving pressure, lung elastance, lung stress and mechanical power compared to 15 cmH_2_O of PEEP.

**Conclusions:**

In COVID-19 ARDS during the early phase the effects of raising PEEP are highly variable and cannot easily be predicted by respiratory system characteristics, because of the heterogeneity of the disease.

**Supplementary Information:**

The online version contains supplementary material available at 10.1186/s13054-021-03839-4.

## Background

The infection with severe acute respiratory syndrome coronavirus 2 (SARS-CoV-2) is characterized by an acute hypoxemic respiratory failure ranging from a mild to a severe form requiring intensive care admission and invasive mechanical ventilation in up of 30% of the severe forms of the disease [1, 2]. Based on the available body of evidence in order to limit the ventilator induced lung injury (VILI), not-related-to-COVID-19-ARDS patients are currently managed by applying low tidal volume without overcoming an inspiratory plateau pressure of 30 cmH_2_O and moderate–high PEEP levels in the moderate–severe forms [3–5]. However, it has also been shown that among ARDS patients similar PEEP levels should not be used, but an individualization is required because the response to PEEP differs according to the respiratory mechanics, hemodynamic, lung recruitability and shunt. Inappropriately too high PEEP levels might promote lung overstress, increase in alveolar dead space and hemodynamic impairment amplifying the VILI [6, 7].

At the present time, no randomized clinical trials which examined the effect of PEEP in COVID-19 ARDS patients (CARDS) have been published [8]. The surviving sepsis campaign on the management of CARDS recommended a higher PEEP strategy rather than lower PEEP strategy [9]. A subsequent consensus of an international panel of experts did not reach any agreement on the PEEP level, suggesting that PEEP should be titrated according to a PEEP/FiO_2_ table or to obtain the best respiratory compliance or the lowest driving pressure [10]. In a large extensive review of the literature the mean applied PEEP level was between 10 and 16 cmH_2_O [8]. However, CARDS patients may present different characteristics from the ARDS, especially in the early phase [11]. CARDS patients may show a discrepancy between a relatively high respiratory system compliance, higher amount of lung gas volume and severity of hypoxemia [11–13]. Lung autopsy in patients who died from CARDS showed a diffuse alveolar damage with significant endotheliitis and microthrombi in the pulmonary vessels [13–16]. In particular, the alteration of perfusion into the lung could be due to an alteration in the hypoxic vasoconstriction (hyperperfusion in the poorly ventilated lung regions) and to a lower perfusion in the aerated lung regions rather than a lung collapse [16]. Consequently, if the vascular derangement is the major mechanism related to hypoxemia, the use of moderate–high PEEP levels is questionable.

Consequently, we hypothesized that as CARDS may present different pathophysiological features than classic ARDS, the application of high levels of end-expiratory pressure is questionable.

Our first aim was to investigate the effects of 5–15 cmH_2_O of PEEP on partitioned respiratory mechanics, gas exchange and dead space; secondly, we investigated whether respiratory system compliance and severity of hypoxemia could affect the response to PEEP on partitioned respiratory mechanics, gas exchange and dead space, dividing the population according to the median value of respiratory system compliance and oxygenation. Thirdly, we explored the effects of an additional PEEP selected according to the Empirical PEEP-FiO_2_ table of the EPVent-2 study on partitioned respiratory mechanics and gas exchange in a subgroup of patients.

## Methods

### Study population

Sixty-one mechanically ventilated patients with a confirmed diagnosis of SARS-CoV-2 admitted to the general intensive care of the ASST Santi Paolo Carlo, Milan, Italy, were enrolled. Inclusion criteria were a laboratory confirmation of SARS-CoV-2 infection based on positive reverse transcriptase–polymerase chain reaction (RT-PCR) assay and diagnosis of ARDS, the day of the study. Exclusion criteria were the presence of barotrauma, the history of severe chronic obstructive pulmonary disease and the hemodynamic instability.

The study was approved by the local ethical committee (Comitato Etico Milano Area I; 2020/ST/095) and informed consent was obtained according to Italian regulations.

### Study protocol

This was an observational study in which two levels of PEEP were tested.

A lung CT scan was performed during an end-expiratory pressure at 5 cmH_2_O of PEEP.

All patients deeply sedated and paralyzed were ventilated in volume control with a tidal volume of 6–8 ml/kg of predicted body weight. Respiratory rate and inspiratory oxygen fraction were selected to maintain a pH and an arterial saturation between 7.34 and 7.44 and 88 and 95%, respectively. A PEEP level of 10 cmH_2_O was clinically set at the beginning in all the patients during the stabilization period (60 min).

Subsequently, a recruitment maneuver was applied in pressure control ventilation with a PEEP of 5 cmH_2_O to reach 45 cmH_2_O of inspiratory plateau pressure with a respiratory rate of 10 for two minutes [17]. Keeping constant the tidal volume, respiratory rate and oxygen fraction, two PEEP levels (5 and 15 cmH_2_O) were selected. Measurements are collected after 20 min of stabilization. The patients underwent the PEEP test in stable hemodynamic conditions. In addition, in a subgroup of 29 patients, the PEEP was adjusted according to the table named Empirical PEEP-FiO_2_ of the EPVent-2 study [18]. The selected PEEP was called the “Empirical PEEP,” and for safety reason it was titrated to limit a maximum of 35 cmH_2_O of inspiratory plateau airway pressure (Additional file 1: Fig. 1S).

### Data collection

Twenty minutes after the application of the selected PEEP arterial/venous blood gas, lung mechanics and hemodynamic were collected. An end-inspiratory and end-expiratory pause were performed to measure the airway and esophageal pressure changes.

#### Gas exchange, respiratory mechanics and lung CT data

In order to measure the esophageal pressure, a radio-opaque balloon catheter (SmartCath Bicore, USA) was positioned in the lowest part of the esophagus and connected to a pressure transducer. The esophageal catheter was inflated of air with 1.5 mL and introduced transorally to reach the stomach at a depth between 50 and 55 cm from the mouth [19]. The intragastric position was confirmed by a rise in intra-abdominal pressure following external manual epigastric compression. Then, it was retracted into the esophagus (i.e., confirmed by the presence of cardiac artifacts in the pressure tracing and by the difference in the absolute pressure) at a distance between 35 and 40 cm from the mouth [19].

During an end-expiratory occlusion by compressing the thorax, the concordant changes of airway and esophageal pressure were verified to check the correct position of the balloon. The amount of gas in the balloon was periodically checked throughout the experiment.

The ventilatory ratio and the estimated physiological dead space were computed according to classic equations (See Additional File 1). The right-to-left intrapulmonary shunt was calculated accordingly to the venous admixture equation, using the blood gas values obtained from the central venous catheter as a surrogate of the mixed venous blood values [20].

The respiratory system, lung, chest wall elastance and lung stress were computed according to the standard formulas (see Additional File 1).

The mechanical power was calculated based on a mathematical simplification of the original mechanical power for volume control-ventilated patients [21]:

$${\text{MP}} = 0.0{98 }*{\text{ Respiratory}}\;{\text{rate }}*{\text{ tidal}}\;{\text{volume [Peak }}\;{\text{ airway}}\;{\text{ Pressure}}{-}\left( {{\text{Plateau}}\;{\text{ airway}}\;{\text{ Pressure}}{-}{\text{PEEP}}} \right) \, /{ 2]}$$

The mechanical power was normalized to the respiratory system compliance [22].

The total lung weight, gas volume and the proportions of the different compartments (not inflated, poorly inflated, well inflated and overinflated) were computed with dedicated software (Maluna) [17].

### Statistical analysis

Continuous data are presented as mean and standard deviation or median and interquartile range, as appropriate, while categorical data are reported as frequencies and percentage. A One-way ANOVA or Friedman test for repeated measure among the levels of PEEP was used to account for the repeated measures design; in case of statistical significance, Holm–Sidak test or Tukey’s test were used, respectively. Characteristics of the patients as well as the differences in respiratory mechanics, gas exchange between the two groups “high PaO_2_/FiO_2_” and “low PaO_2_/FiO_2_” or between “high Compliance” or “low Compliance” were compared by the Student’s t-test or Wilcoxon–Mann–Whitney rank sum test, as appropriate. The chi-square test or Fisher’´s exact test of independence to analyze for frequency count. A *p* value of 0.05 or less was considered statistically significant. The statistical analysis was performed with SigmaPlot 11.0 (Systat Software, San Jose, CA) and RStudio (R Foundation for Statistical Computing, Vienna, Austria).

## Results

The main characteristics of the patients are presented in Additional file 1: Table 1S. The days between the onset of symptoms and hospital admission were 6 [5–8]. The median PaO_2_/FiO_2_ at baseline was 113 [79–158] mmHg (15.1 [10.5–21.1] kPa). Thirty-eight patients (62%) and 19 patients (31%) presented a severe and moderate form of CARDS. The mean lung gas volume and weight were 1441 [923–2235] mL and 1348 [946–1647] g, respectively; the amount of not aerated tissue was 10.7 [3.1–19.1] % of the total lung weight (Additional file 1: Table 1S).

### *Response to 5–15 cmH*_*2*_*O of PEEP on partitioned respiratory mechanics, gas exchange and dead space*

**Fig. 1:**
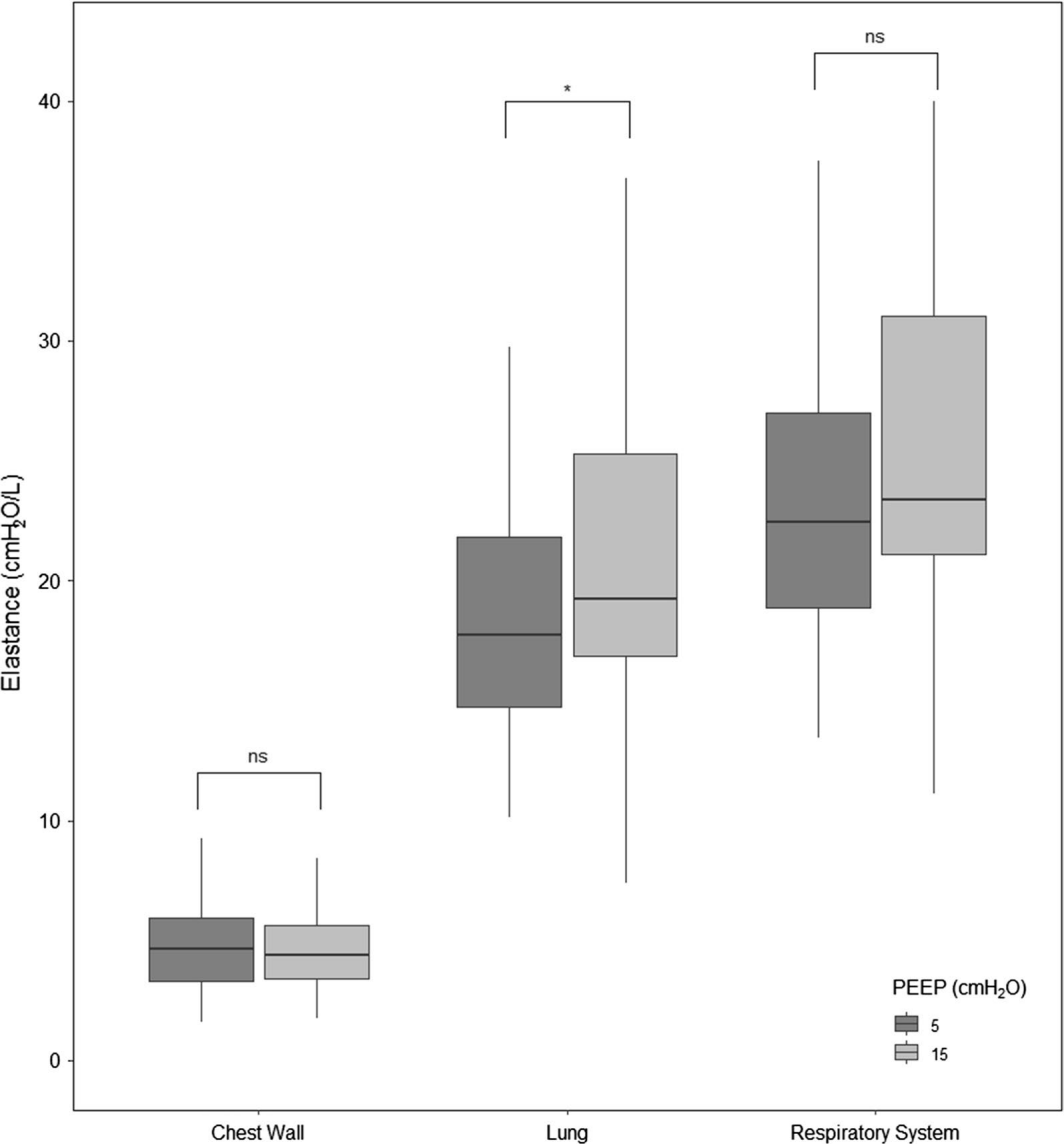
Respiratory system, chest wall and lung elastance at 5 and 15 cmH_2_O of PEEP of the whole population. *p < 0.05; ns: not significant

In Table 1 the data of the respiratory mechanics and gas exchange at 5 and 15 cmH_2_O of PEEP are presented. At 15 cmH_2_O of PEEP the lung elastance, lung stress and mechanical power were significantly higher compared to 5 cmH_2_O. Although the airway plateau pressure was significantly higher at 15 cmH_2_O, the driving pressure and elastance of respiratory system increased but did not reach statistical significance (Fig. 1; Additional file 1: Fig. 2S).

**Table 1:** Respiratory mechanics and gas exchange within 5 and 15 cmH_2_O of PEEP

Variables	PEEP 5 61 patients	PEEP 15 61 patients	*p*
Plateau pressure, cmH_2_O	17 ± 3	28 ± 3	**< *0.001***
Driving pressure, cmH_2_O	12 ± 3	13 ± 3	*0.071*
Respiratory system elastance, cmH_2_O/L	23 [19–27]	24 [21–31]	*0.093*
Lung elastance, cmH_2_O/L	18 [15–22]	19 [17–25]	***0.049***
Chest wall elastance, cmH_2_O/L	5 [3–6]	4 [3–6]	*0.845*
Lung stress, cmH_2_O	13.1 ± 3.1	22.3 ± 4.0	** < *0.001***
Mechanical power, J/min	16.7 ± 5.7	26.1 ± 6.4	** < *0.001***
Mechanical Power_Compliance_rs_, J/min/(mL/cmH_2_O)	0.36 [0.27–0.50]	0.61 [0.51–0.77]	** < *0.001***
PaO_2_, mmHg	63 [55–76]	84 [74–99]	** < *0.001***
PaO_2_/FiO_2_, mmHg	82 [66–138]	127 [90–184]	** < *0.001***
Right-to-left shunt, %	47 ± 15	35 ± 12	** < *0.001***
PvO_2_, mmHg	42 ± 7	47 ± 7	** < *0.001***
ScvO_2_, %	74 ± 8	79 ± 6	** < *0.001***
C_a-v_O_2_, mL	2.8 [2.3–3.2]	3.0 [2.4–3.4]	***0.024***
PaCO_2_, mmHg	48 ± 9	50 ± 9	** < *0.001***
Ventilatory ratio	1.66 ± 0.39	1.74 ± 0.37	***0.002***
Estimated physiological dead space	0.50 [0.43–0.58]	0.52 [0.42–0.60]	** < *0.001***

Paired T-test or Wilcoxon–Mann–Whitney test for repeated measures were performed, as appropriate. Tidal volume and respiratory rate were unchanged between the two PEEP levels. C_a-v_O_2_: arterial–venous oxygen content difference; Compliance_rs_: respiratory system compliance; ScvO_2_ central oxygen venous saturation; PvO_2_ mixed venous oxygen tension

Concerning gas exchange, the PaO_2_/FiO_2_, arterial carbon dioxide and ventilatory ratio significantly increased at 15 cmH_2_O of PEEP. The arterial–venous oxygen difference and central venous saturation were significantly higher at 15 cmH_2_O of PEEP. The shunt significantly decreased at 15 cmH_2_O of PEEP. Increasing PEEP from 5 to 15 cmH_2_O the mechanics and gas exchange variables that significantly increased showed high heterogeneity.

### *Response to 5–15 cmH*_*2*_*O of PEEP according to the compliance of respiratory system and severity of hypoxemia*

Considering the median of respiratory compliance of the whole population (44 ml/cmH_2_O) to separate the population in a high and a low compliance group, the first group had a higher lung gas volume and well inflated lung tissue (Additional file 1: Table 2S; Fig. 3S). The oxygenation and ventilatory ratio were not different (Additional file 1: Table 2S).

By increasing the PEEP from 5 to 15 cmH_2_O, the changes in the partitioned respiratory system elastance and gas exchange were similar between the two groups (Table 2, Additional file 1: Fig. 5S).

**Table 2:** Changes in respiratory mechanics and gas exchange at 5 and 15 cmH_2_O of PEEP between high and low respiratory system compliance group

Variables	Low C_rs_ < 44 mL/cmH_2_O 30 patients	High C_rs_ ≥ 44 mL/cmH_2_O 31 patients	*p*
Δ_15-5_ Plateau pressure, cmH_2_O	10 [7–12]	11 [10–13]	***0.042***
Δ_15-5_ Driving pressure, cmH_2_O	0 [− 3 to 2]	1 [0–3]	***0.042***
Δ_15-5_ Respiratory system elastance, cmH_2_O/L	− 1 [− 5 to 4]	2 [0–6]	*0.057*
Δ_15-5_ Lung elastance, cmH_2_O/L	2 [− 2 to 5]	2 [0–5]	*0.286*
Δ_15-5_ Chest wall elastance, cmH_2_O/L	0 [− 2 to 2]	0 [− 1 to 0]	*0.305*
Δ_15-5_ Lung stress, cmH_2_O	9 [6–11]	9 [8–12]	*0.256*
Δ_15-5_ Mechanical power, J/min	9 [8–12]	9 [8–11]	*0.866*
Δ_15-5_ Mechanical Power_Compliance_rs_, J/min/(mL/cmH_2_O)	0.26 [0.12–0.40]	0.23 [0.16–0.35]	*0.656*
Δ_15-5_ PaO_2_, mmHg	20 [13–38]	20 [6–30]	*0.336*
Δ_15-5_ PaO_2_/FiO_2_, mmHg	28 [16–51]	30 [10–50]	*0.657*
Δ_15-5_ Right-to-left shunt, %	− 12 [− 23 to − 1]	− 9 [− 18 to − 5]	*0.710*
Δ_15-5_ PvO_2_, mmHg	4.0 [2.3–8.8]	2.0 [1.0–8.1]	*0.219*
Δ_15-5_ ScvO_2_, %	5.5 [1.0–10.8]	2.5 [0.0–8.1]	*0.184*
Δ_15-5_ C_a-v_O_2_, mL	1.0 [0.3–1.7]	0.6 [0.2–1.3]	*0.331*
Δ_15-5_ PaCO_2_, mmHg	1 [− 1 to 3]	2 [1–4]	*0.226*
Δ_15-5_ Ventilatory ratio	0.04 [− 0.10 to 0.10]	0.08 [0.02–0.20]	*0.119*
Δ_15-5_ Estimated physiological dead space	0.01 [− 0.01 to 0.03]	0.02 [0.01–0.05]	*0.119*

T-test or Wilcoxon–Mann–Whitney test were performed, as appropriate. Δ_15-5_: difference between 15 and 5 cmH_2_O of PEEP; C_a-v_O_2_: arterial–venous oxygen content difference; Compliance_rs_: respiratory system compliance; ScvO_2_ central oxygen venous saturation; PvO_2_ mixed venous oxygen tension

Furthermore, we also divided the whole population according to the median of the PaO_2_/FiO_2_ (81.8 mmHg or 10.9 kPa), in high and low PaO_2_/FiO_2_ groups. The two groups presented similar lung gas volume, not inflated tissue and partitioned respiratory mechanics (Additional file 1: Table 3S; Fig. 4S). By increasing the PEEP from 5 to 15 cmH_2_O, the changes in partitioned respiratory mechanics and mechanical power were similar (Table 3, Additional file 1: Fig. 6S).

**Table 3:** Changes in respiratory mechanics and gas exchange at 5 and 15 cmH_2_O of PEEP between high and low PaO_2_/FiO_2_ group

Variables	Low PaO_2_/FiO_2_ < 81.8 31 patients	High PaO_2_/FiO_2_ ≥ 81.8 30 patients	*p*
Δ_15-5_ Plateau pressure, cmH_2_O	8 [10–11]	11 [10–13]	*0.147*
Δ_15-5_ Driving pressure, cmH_2_O	0 [− 2 to 1]	1 [0–3]	*0.147*
Δ_15-5_ Respiratory system elastance, cmH_2_O/L	0 [− 3 to 3]	3 [0–6]	*0.126*
Δ_15-5_ Lung elastance, cmH_2_O/L	2 [− 2 to 4]	3 [1–7]	*0.257*
Δ_15-5_ Chest wall elastance, cmH_2_O/L	0 [− 2 to 1]	0 [− 2 to 2]	*0.373*
Δ_15-5_ Lung stress, cmH_2_O	9 [7–11]	9 [8–13]	*0.534*
Δ_15-5_ Mechanical power, J/min	9 [8–11]	10 [9–11]	*0.351*
Δ_15-5_ Mechanical Power_Compliance_rs_, J/min/(mL/cmH_2_O)	0.21 [0.12–0.34]	0.30 [0.17–0.37]	*0.436*
Δ_15-5_ PaO_2_, mmHg	26 [16–38]	16 [4–23]	***0.012***
Δ_15-5_ PaO_2_/FiO_2_, mmHg	31 [16–55]	28 [9–42]	*0.212*
Δ_15-5_ Right-to-left shunt, %	− 16 [− 26 to − 10]	0 [− 11 to 0]	** < *0.001***
Δ_15-5_ PvO_2_, mmHg	3 [2–11]	1 [0–2]	** < *0.001***
Δ_15-5_ ScvO_2_, %	8.5 [4.4–13.4]	0.6 [− 1 to 3]	** < *0.001***
Δ_15-5_ C_a-v_O_2_, mL	1.3 [0.9–1.8]	0.4 [0.0–0.6]	** < *0.001***
Δ_15-5_ PaCO_2_, mmHg	1 [− 2 to 3]	2 [1–5]	*0.110*
Δ_15-5_ Ventilatory ratio	0.04 [− 0.09 to 0.10]	0.08 [− 0.02 to 0.19]	*0.065*
Δ_15-5_ Estimated physiological dead space	0.01 [− 0.02 to 0.03]	0.02 [0.01–0.05]	*0.070*

T-test or Wilcoxon–Mann–Whitney test were performed, as appropriate. Δ_15-5_: difference between 15 and 5 cmH_2_O of PEEP; C_a-v_O_2_: arterial–venous oxygen content difference; Compliance_rs_: respiratory system compliance; ScvO_2_ central oxygen venous saturation; PvO_2_ mixed venous oxygen tension

### Response to Empirical PEEP on partitioned respiratory mechanics, gas exchange and dead space

In a subgroup of 29 patients the Empirical PEEP has been selected according to the PEEP/FIO_2_ table. The Empirical PEEP was 18 ± 1 cmH_2_O. The Empirical PEEP significantly increased the driving pressure, elastance, lung stress and mechanical power compared to 15 cmH_2_O of PEEP (Additional file 1: Table 4S; Fig. 7S). At the Empirical PEEP the PaO_2_/FiO_2_ was higher compared to 15 cmH_2_O of PEEP, while carbon dioxide and ventilatory ratio were similar.

## Discussion

The main findings of this study evaluating different PEEP levels, with constant tidal volume, were: (1) 15 cmH_2_O of PEEP significantly increased the ventilatory ratio, lung elastance and mechanical power although with heterogeneous responses, (2) the arterial oxygenation increased by increasing the PEEP, (3) the compliance of the respiratory system and the level of hypoxemia at baseline did not affect the PEEP response and (4) the PEEP suggested by the PEEP/FiO_2_ table was higher than 15 cmH_2_O.

Most mechanically ventilated CARDS patients fulfill the criteria of ARDS according to the Berlin definition [23, 24]. Typically, ARDS is characterized by an inflammatory pulmonary edema, shunt related hypoxemia and reduction both in lung gas volume and in the respiratory compliance [6]. Higher PEEP levels, recruitment maneuvers and prone positioning are suggested to recruit the lung [4, 7]. However, COVID-19 is a systemic disease which besides affecting mainly the lung, can also damage the vascular endothelium [13, 17]. It has been reported an activation of the coagulation cascade with an associated micro–macro thrombosis in the lung. It has also been found the presence of vessels enlargement in the ground glass opacities, suggesting the presence of a thrombotic inflammatory process [16]. The available data clearly indicate that CARDS can present heterogeneous characteristics with a relatively high respiratory system compliance [11, 12, 25–27]. Our group proposed the presence of two phenotypes in CARDS based on lung mechanics properties, type L (low elastance) and type H (high elastance) [28, 29]. In the present study our population had a median of 2 [2] days of mechanical ventilation since intubation and the PEEP trial, presented a median compliance of respiratory system of 44 ml/cmH_2_O. Similar data were also reported by other groups [25, 30, 31]. In a recent review, the compliance values at 1 SD above the mean or the 75th percentile > 50 ml/cmH_2_O were reported in 43% of the studies [8]. Similarly, the lung gas volume computed by CT was also considerable high, a median of 2952 [2038–3617] mL, contrary to previous data on ARDS patients in which the lung gas volume (i.e., baby lung) was typically reduced [17, 32, 33].

In a recent review analyzing the available data about mechanical ventilation setting in CARDS, it was reported that PEEP values ranged from 9 to 16.5 cmH_2_O [8]. The PEEP was selected mainly according to the change in oxygenation or to a PEEP/FiO_2_ table [34, 35].

### *Response to 5–15 cmH*_*2*_*O of PEEP on partitioned respiratory mechanics, gas exchange and dead space*

In this study we choose to evaluate the effects of two different PEEP levels (5 and 15 cmH_2_O) based on previous studies which defined as “low” and “moderate–high” PEEP [17, 36]. The higher PEEP levels significantly increased the oxygenation and reduced the alveolar shunt. However, at the same time, in the majority of patients the ventilatory ratio was higher indicating a less efficiently CO_2_ clearance (*i.e.,* higher dead space) as well as the respiratory system compliance decreased. Similar data was also found by Mauri et al. in a small group of CARDS (10 patients), in which 15 cmH_2_O of PEEP was associated with a better oxygenation and higher arterial carbon dioxide [25]. In patients with ARDS it has been observed that increasing PEEP the arterial oxygenation increased by a reduction in the intrapulmonary shunt with also an increase in the dead space [37].

More importantly, in our patients the increase of PEEP significantly increased the arterial–venous difference in oxygenation and the central venous oxygenation.

Thus, the ameliorating in oxygenation at higher PEEP levels seems more related to the modification of the ventilation/perfusion ratio in the lung areas with low ventilation/perfusion rather than a lung recruitment (i.e., reopening of collapsed lung regions) [11]. The reduction in the alveolar shunt could also be due to a decrease in cardiac output by the higher levels of PEEP [37–39].

Although the plateau airway pressure and driving pressure are commonly used as surrogate of the VILI, the real distending force of the lung is the transpulmonary pressure [4, 40, 41]. The transpulmonary pressure computed by the esophageal balloon technique is closely related to the lung stress [41, 42]. Thus, in the present study we assessed both the lung elastance and the lung stress. The enrolled patients have a relatively normal chest wall elastance which contributes to the elastance of the respiratory system by approximately 20–30% [41]. By note, at higher PEEP levels the lung elastance and lung stress were significantly higher. In a previous small study, applying the electrical impedance tomography technique (EIT), higher PEEP levels were associated with higher lung elastance and to higher lung overdistension evaluated by EIT technique [43]. In addition to evaluate the overall effect of different levels of PEEP, we computed the mechanical power which depends on the driving pressure, tidal volume, respiratory rate and PEEP. The respiratory rate and tidal volume did not change throughout the study; thus, the mechanical power resulted from the interaction of PEEP and driving pressure. By increasing PEEP, the mechanical power and the normalized mechanical power for the compliance of respiratory system (i.e., to take into account the size of the lung) were much higher compared to low PEEP level.

### *Response to 5–15 cmH*_*2*_*O of PEEP according to the compliance of respiratory system and severity of hypoxemia*

In ARDS patients several studies evaluated the possible factors associated with PEEP response [4, 6]. We found a comparable median response to PEEP in both patients with a low and high respiratory system compliance and with high and low PaO_2_/FiO_2_ in whom the oxygenation improved, the mechanical power significantly increased while the ventilatory ratio and respiratory mechanics, although deteriorating, showed high heterogeneity. Perier et al., comparing two CARDS subgroups based on the compliance of the respiratory system assessed at low PEEP level, found a similar behavior in terms of collapse, hyperdistention and oxygenation by applying a range of PEEP from 6 to 18 cmH_2_O [44]. In our population, the more hypoxemic group, which seems to improve the oxygenation when PEEP was increased from 5 to 15 cmH_2_O, showed the same quantitative radiological features at the CT scan at 5 cmH_2_O compared with less hypoxemic group. This can partially explain that the changes in the partitioned respiratory system elastance and gas exchange were similar between the two groups. Similarly, the group with low and high respiratory system compliance presented the same amount of not and poorly inflated tissue, with similar changes in the partitioned respiratory system elastance and gas exchange between the two groups.

### Response to Empirical PEEP on partitioned respiratory mechanics, gas exchange and dead space

In ARDS patients the use of PEEP-FiO_2_ tables is a quite common approach in clinical practice mainly due to the greater ease of application [4]. Several PEEP-FiO_2_ tables have been proposed over the decades [4, 6]. In two ARDS randomized clinical trials that compared a lower and higher PEEP-FiO_2_ table, the arterial oxygenation increased with the higher PEEP table, suggesting a higher lung recruitment a better outcome [6]. In ARDS patients the PEEP-FiO_2_ table was able to provide PEEP levels according to the lung recruitability [7]. In the present study applying a PEEP-FiO_2_ combination table commonly suggested in ARDS, the proposed average PEEP level was 18 ± 1 cmH_2_O. However, this Empirical PEEP level was associated with a significantly higher oxygenation but to a worsening in ventilatory ratio and lung elastance compared to 15 cmH_2_O. Tsolaki et al., applying the ARDS net protocol, according to the Surviving sepsis campaign, found that the suggested PEEP level was 18 cmH_2_O instead of a mean level of 8 cmH_2_O of PEEP, when the best combination of respiratory system compliance, CO_2_ clearance and hemodynamic was used [45]. In a small study of 15 CARDS comparing the PEEP selected by the EIT technique to obtain to the best compromise between lung collapse and overdistension, with PEEP/FiO_2_ table approach, the latter suggested higher PEEP (17 vs 12 cmH_2_O) [46]. Thus, if we followed in CARDS the Empirical PEEP-FiO_2_ table, significantly higher PEEP levels should have been applied in lungs with almost normal compliance with possible detrimental consequences.

This study has several strengths: Firstly, it is the first study which computed the lung compliance and mechanical power during the change of PEEP; secondly, it evaluated two PEEP levels (low and moderate/high) compared to an Empirical PEEP-FiO_2_ table, which is suggested in ARDS patients.

### Limitations

The possible limitations of this study were the absence of any data regarding the computation of lung recruitability at 15 cmH_2_O of PEEP and regarding the changes of cardiac output at the different PEEP levels.

## Conclusions

In conclusion, the main finding of this study evaluating different PEEP levels with constant tidal volume was that the effects of raising PEEP are highly variable among CARDS patients and cannot easily be predicted by respiratory system characteristics, because of the heterogeneity of the disease, including with respect to respiratory system compliance and hypoxia that is mainly due to low V/Q respond less than to true shunt. Moreover, the PEEP adjusted according to the Empirical PEEP-FiO_2_ table resulted in an unnecessary much higher PEEP levels possibly which was not related to the pathophysiology of CARDS patients. Moderate PEEP levels are able to redistribute the pulmonary blood flow, ameliorate oxygenation and avoid the lung over stress and strain and the impairment of cardiac function with a higher need of fluids and vasopressor. Thus, in CARDS tailoring PEEP based on a “PEEP test,” represents an invaluable option to set a protective lung ventilation and prevent further damage to the lungs.

## Supplementary Information

**Additional file 1**. Respiratory mechanics and physiological variables formulas; study protocol flow-chart; baseline characteristics table; additional data about PEEP 5-15cmH2O comparison; boxplot and linear regression graphs.

## Data Availability

The dataset used in writing this manuscript are available from the corresponding author after reasonable request.

## Abbreviations

ARDS: Acute respiratory distress syndrome
CARDS: COVID19 ARDS
COVID19: Coronavirus disease 2019
FiO2: Fraction of inspired oxygen
EIT: Electrical impedance tomography technique
PEEP: Positive end-expiratory pressure
SARS-CoV-2: Severe acute respiratory syndrome coronavirus-2
