# Supplementary material for: Positive end-expiratory pressure in COVID-19 acute respiratory distress syndrome: the heterogeneous effects
Source: Crit Care. 2021 Dec 16;25:431. doi: 10.1186/s13054-021-03839-4 (PMC8674862; doi:10.1186/s13054-021-03839-4)
Supplement: Supplementary file 1 — Additional file 1. Respiratory mechanics and physiological variables formulas; study protocol flow-chart; baseline characteristics table; additional data about PEEP 5-15cmH2O comparison; boxplot and linear regression graphs. [file 13054_2021_3839_MOESM1_ESM.docx]

**Online Supplementary Material**

**Bedside selection of positive end expiratory pressure in COVID-19 acute respiratory distress syndrome**

Davide Chiumello MD^1,2,3^, Matteo Bonifazi MD^1^, Tommaso Pozzi MD^1^, Paolo Formenti MD^1^, Giuseppe Francesco Sferrazza Papa, Gabriele Zuanetti MD^2^, Silvia Coppola MD^1^

[1]

$$Driving Pressure \left( cmH_{2}O \right)=Plateau airway pressure-PEEP$$

[2]

$$Respiratory System Elastance (\frac{cmH_{2}O}{L})=\frac{Driving Pressure (cmH_{2}O)}{Tidal Volume (mL)}$$

[3]

$$Chest Wall Elastance (\frac{cmH_{2}O}{L})=\frac{Plateau Esophageal Pressure-End Expiratory Esophageal Pressure}{Tidal Volume (mL)}$$

[4]

$$Lung Elastance \left( \frac{cmH_{2}O}{L} \right)=Respiratory System Elastance \left( \frac{cmH_{2}O}{L} \right)-Chest Wall Elastance (\frac{cmH_{2}O}{L})$$

[5]

$$Lung Stress \left( cmH_{2}O \right)=Plateau Pressure \left( cmH_{2}O \right)\times\frac{Lung Elastance}{Respiratory System Elastance}$$

Chiumello D, Cressoni M, Colombo A, et al. The assessment of transpulmonary pressure in mechanically ventilated ARDS patients. *Intensive Care Med*. 2014;40(11):1670-1678.

[6]

$$Ventilatory Ratio=\frac{{Minute Ventilation}_{measured}\left[ \frac{mL}{min} \right]\times{PaCO}_{2 measured} [mmHg]}{{Minute Ventilation}_{predicted}\left[ \frac{mL}{min} \right] \times{PaCO}_{2 ideal} [mmHg]}$$

[7]

$${Minute Ventilation}_{predicted}=Predicted Body Weight \times100 \left[ \frac{mL}{min} \right]$$

[8]

$${PaCO}_{2 ideal}=37.5 mmHg$$

Sinha P, Calfee CS, Beitler JR, Soni N, Ho K, Matthay MA, Kallet RH. Physiologic Analysis and Clinical Performance of the Ventilatory Ratio in Acute Respiratory Distress Syndrome. Am J Respir Crit Care Med. 2019 Feb 1;199(3):333-341.

[9]

$$Estimated Physiological Dead Space=1-\frac{0.863\times{\dot{V}CO}_{2}}{Respiratory Rate [bpm]\times Tidal Volume [L]\times{PaCO}_{2 measured} [mmHg]}$$

Where VCO_2_ represents CO_2_ production (mL/min).

[10]

$${\dot{V}CO}_{2}=\frac{Resting Energy Expenditure}{\frac{5.616}{0.8}}+1.584$$

[11]

${Resting Enery Expenditure}_{Unadjusted Harris-Benedict Estimate-Male}=66.473 + 13.752 \times Weight \left[ kg \right]+5.003\times Height \left[ cm \right]- 6.755\times Age \left[ years \right]$

[12]

$${Resting Enery Expenditure}_{Unadjusted Harris-Benedict Estimate-Female}=655.096 + 9.563 \times Weight \left[ kg \right]+1.850\times Height \left[ cm \right]- 4.676\times Age \left[ years \right]$$

Morales-Quinteros L, Schultz MJ, Bringué J, Calfee CS, Camprubí M, Cremer OL, Horn J, van der Poll T, Sinha P, Artigas A, Bos LD; MARS Consortium. Estimated dead space fraction and the ventilatory ratio are associated with mortality in early ARDS. Ann Intensive Care. 2019 Nov 21;9(1):128.

[13]

$$Right-to-left shunt=\frac{CcO_{2}-CaO_{2}}{CcO_{2}-CvO_{2}}$$

Where CcO_2_ is the capillary oxygen content, CaO_2_ is the arterial oxygen content and CvO_2_ is the venous oxygen content.

[14]

$$CcO_{2}=1.36\times\left[ Hb \right]+0.0031\times P_{A}O_{2}$$

Where [Hb] is the blood hemoglobin concentration (g/dL), SaO_2_ is the arterial oxygen hemoglobin saturation and P_A_O_2_ is the alveolar oxygen partial pressure (mmHg).

[15]

$$P_{A}O_{2}=\left( Pbar-P_{H_{2}o} \right)\times FiO_{2}-\frac{Pa{CO}_{2}}{RQ}$$

Where Pbar is the atmospheric pressure (estimated to be 760 mmHg), pH_2_O is the water vapour pressure (estimated to be 47 mmHg), FiO_2_ is the inspired fraction of oxygen, PaCO_2_ is the arterial carbon dioxide partial pressure (mmHg) and RQ is the respiratory quotient, estimated to be 0.8 in our population.

[16]

$$CaO_{2}=1.36\times\left[ Hb \right]\times SaO_{2}+0.0031\times P_{a}O_{2}$$

Where SaO_2_ is the arterial oxygen-hemoglobin saturation and PaO_2_ is the arterial oxygen partial pressure (mmHg).

[17]

$$CvO_{2}=1.36\times\left[ Hb \right]\times ScvO_{2}+0.0031\times P_{v}O_{2}$$

Where ScvO_2_ is the central venous oxygen-hemoglobin saturation and PvO_2_ is the central venous oxygen partial pressure (mmHg).

Gattinoni L, Caironi P, Cressoni M, et al. Lung recruitment in patients with the acute respiratory distress syndrome. *N Engl J Med*. 2006;354(17):1775-1786.

**Figure 1S**. Flow chart of the study protocol.


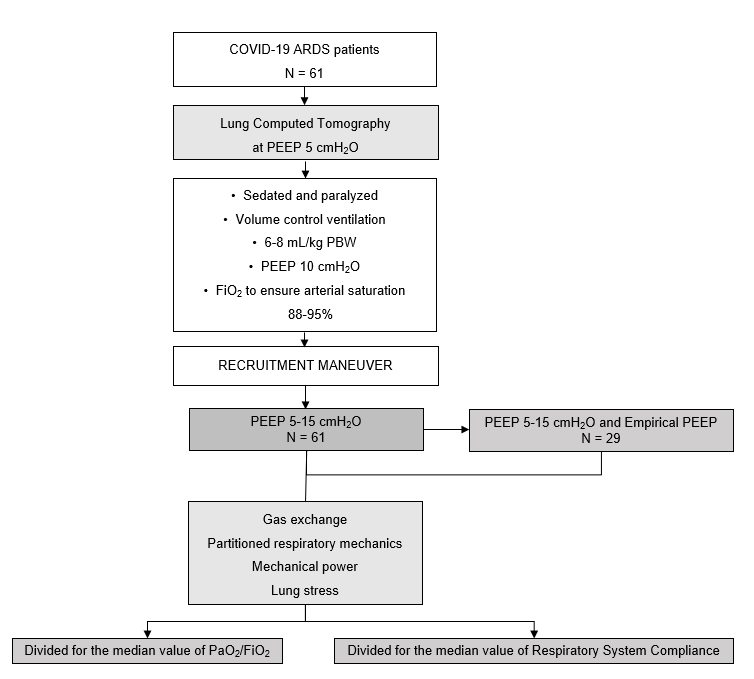


**Table 1S.** Baseline characteristics of the study population. Quantitative data are expressed as median [IQR], while categorical data are expressed as % (n).

| **Baseline variables** | **STUDY POPULATION**  **61 patients** |  |
| --- | --- | --- |
| Age, *years* | 60 [54 – 67] |  |
| Male sex, % (n) | 85 (52) |  |
| Body Mass Index, *kg/m^2^* | 27 [26 – 33] |  |
| SAPS II | 33 [28 – 37] |  |
| Time between ETI and study day, *days* | 2 [2 – 2] |  |
| Tidal volume/PBW, *mL/kg* | 7.2 [6.8 - 7.7] |  |
| Respiratory rate, *bpm* | 18 [16-19] |  |
| Minute ventilation, *L/min* | 8.8 [8.2-9.8] |  |
| PEEP, *cmH_2_O* | 10 [10 - 10] |  |
| Plateau pressure, *cmH_2_O* | 21.5 [20-23] |  |
| Driving pressure, *cmH_2_O* | 11 [10-13] |  |
| Respiratory system compliance, *mL/cmH_2_O* | 44 [37 – 52] |  |
| Mechanical power, *J/min* | 19 [17 – 22] |  |
| Mechanical Power_Compliance_rs_, *J/min/(mL/cmH_2_O)* | 0.36 [0.27 – 0.5] |  |
| Arterial pH | 7.38 [7.34-7.44] |  |
| PaO_2_, *mmHg* | 81.3 [68.9-90.5] |  |
| PaO_2_/FiO_2_, *mmHg* | 113.7 [79.3 - 158] |  |
| Right-to-left shunt, *%* | 47 [38 – 56] |  |
| PvO_2_, *mmHg* | 43 [38 – 47] |  |
| ScvO_2_, *%* | 74 [71 – 79] |  |
| C_a-v_O_2_, *mL* | 2.8 [2.4 – 3.3] |  |
| PaCO_2_, *mmHg* | 47 [41 - 52] |  |
| Ventilatory ratio | 1.57 [1.36-1.85] |  |
| Estimated physiological dead space | 0.50 [0.43 – 0.58] |  |
| Outcome, *% (n)*  Dead  Alive | 46 (28)  54 (33) |  |
| Total lung weight, *g* | 1343 [948 - 1647] |  |
| Total gas volume, *mL* | 1441 [923 – 2235] |  |
| Total lung volume, *mL* | 2952 [2038 – 3617] |  |
| Over inflated tissue, *g* | 3.3 [0.8-12.8] |  |
| Well inflated tissue, *g* | 438 [306-556] |  |
| Poorly inflated tissue, *g* | 455 [299-619] |  |
| Not inflated tissue, *g* | 310 [128-600] |  |
| Over inflated tissue, *%* | 1.9 [0.5 - 7.2] |  |
| Well inflated tissue, *%* | 60.2 [48.1 – 68.0] |  |
| Poorly inflated tissue, *%* | 21.5 [15.8-28.4] |  |
| Not inflated tissue, *%* | 10.7 [3.1 – 19.1] |  |

SAPS II: Simplified Acute Physiology Score; C_a-v_O_2_: arterial-venous oxygen content difference; Compliance_rs_: respiratory system compliance; ScvO_2_ central oxygen venous saturation; PvO_2_ mixed venous oxygen tension.

**Figure 2S.** Boxplot and two-point graphical representation showing each patient’s variable at 5 and 15 cmH_2_O of PEEP.

| 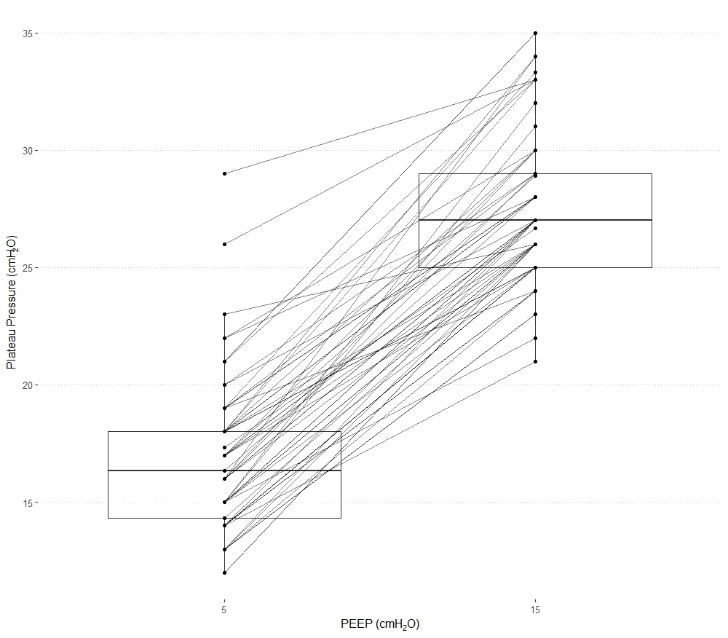 | 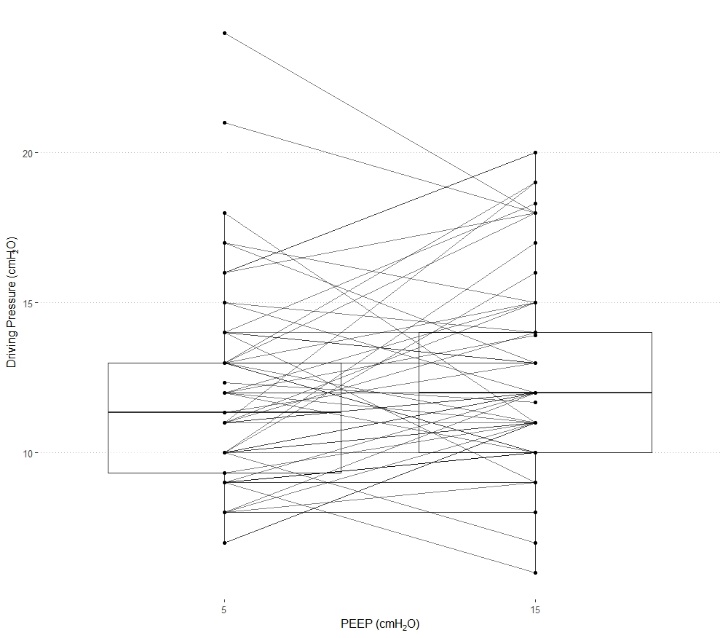 |
| --- | --- |
| 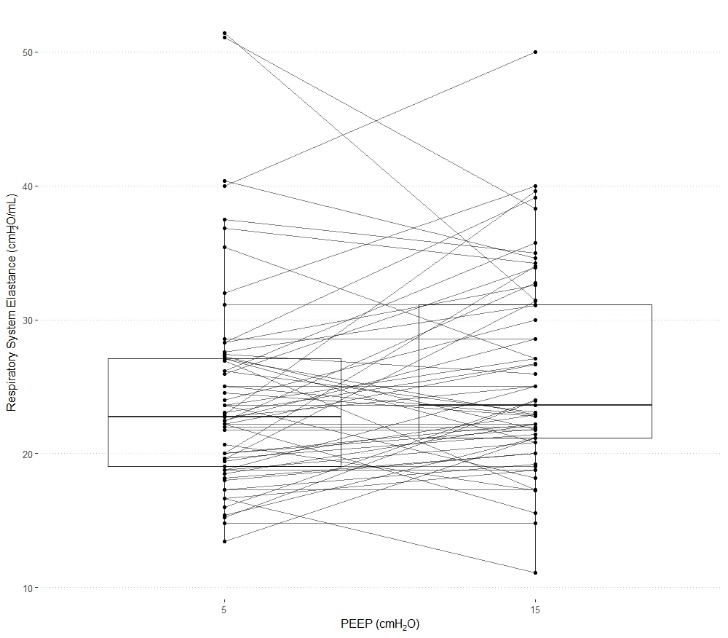 | 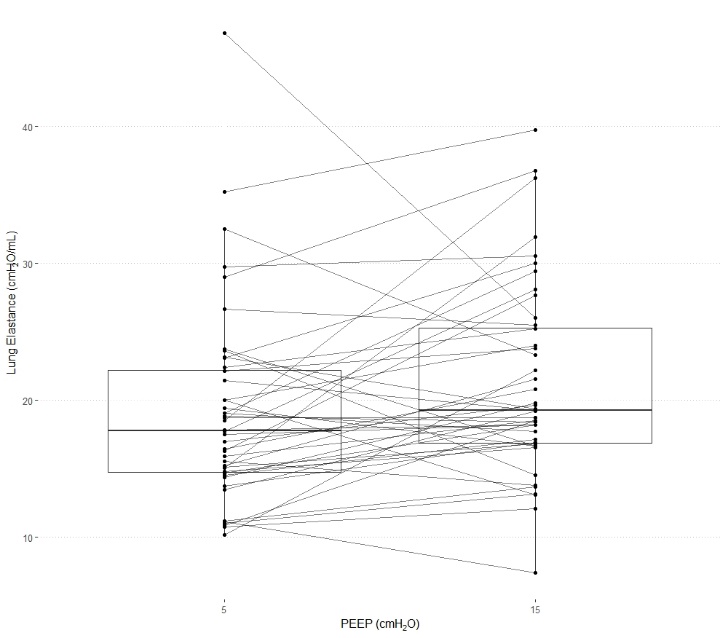 |
| 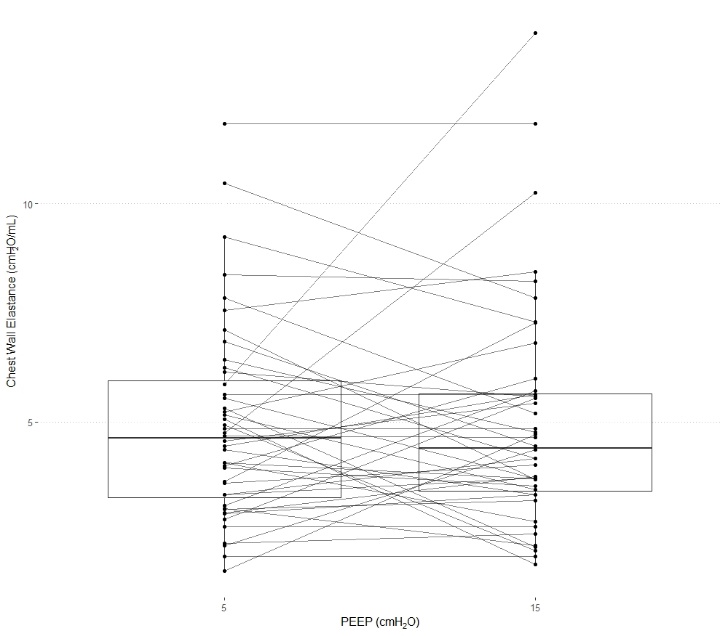 | 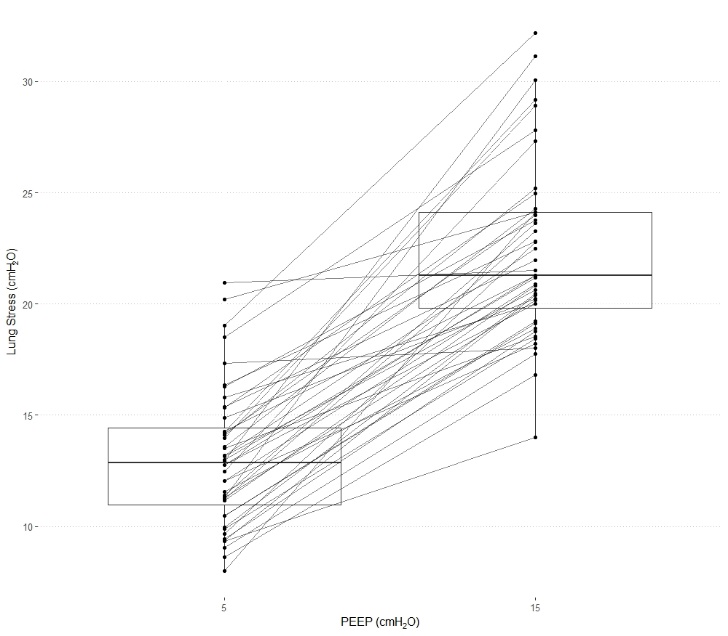 |
| 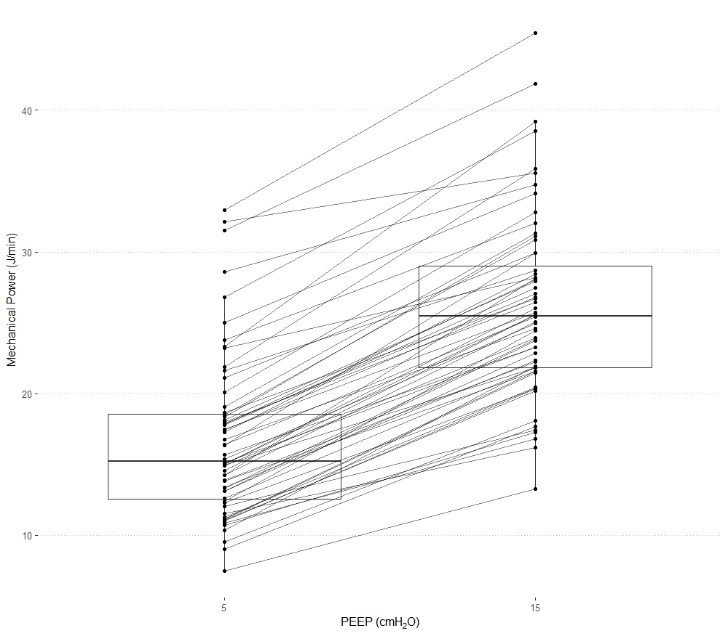 | 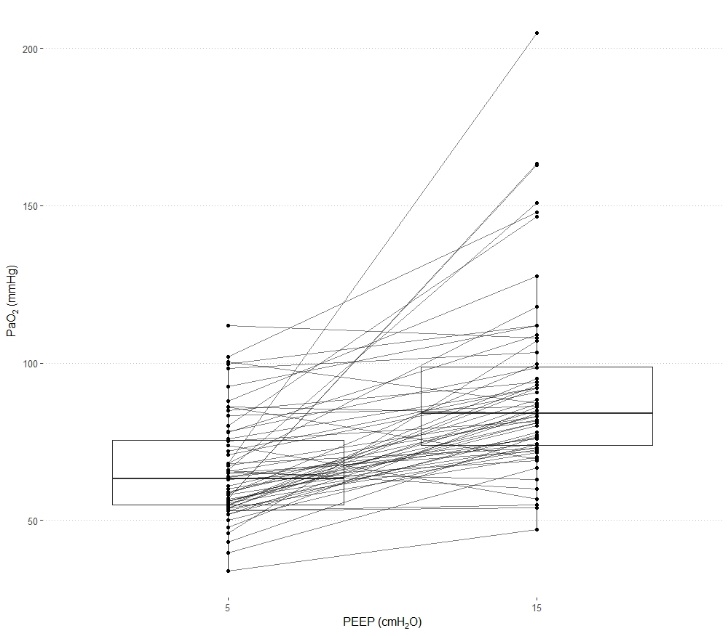 |
| 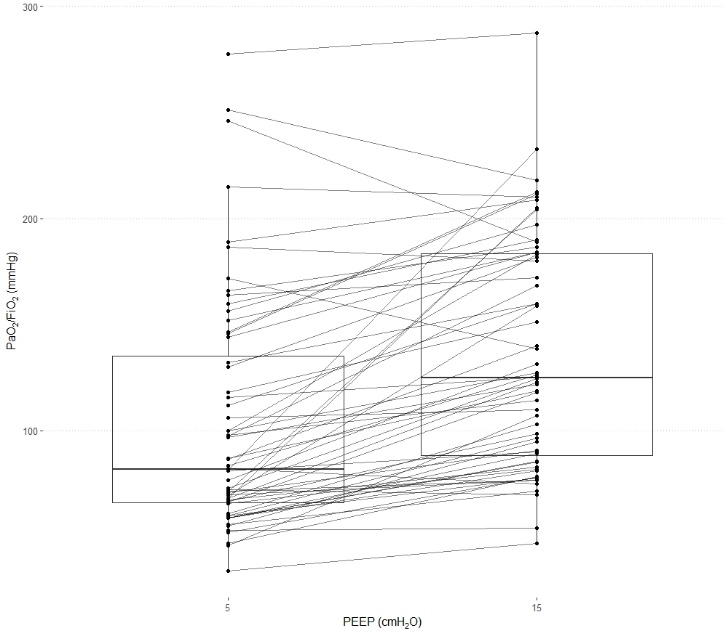 | 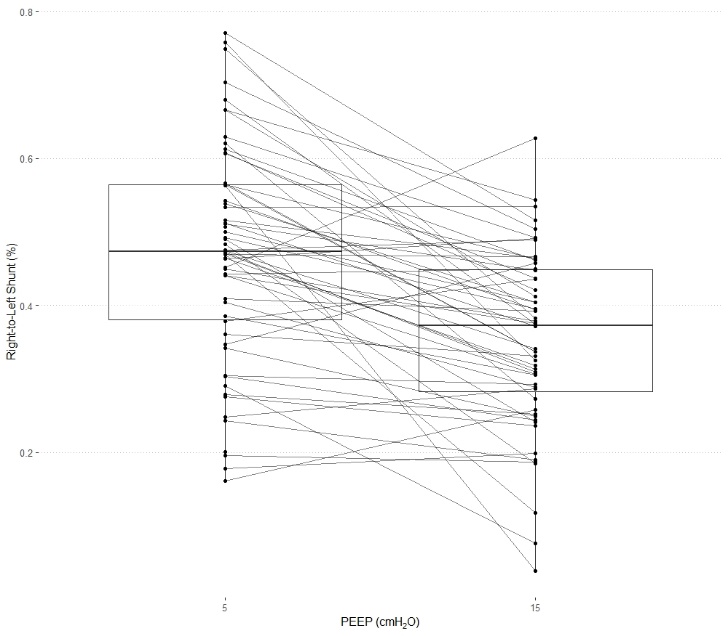 |
| 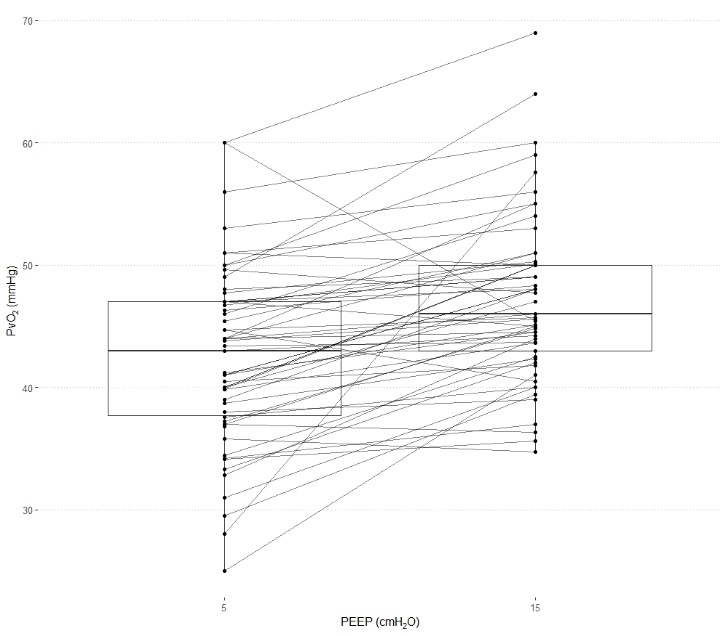 | 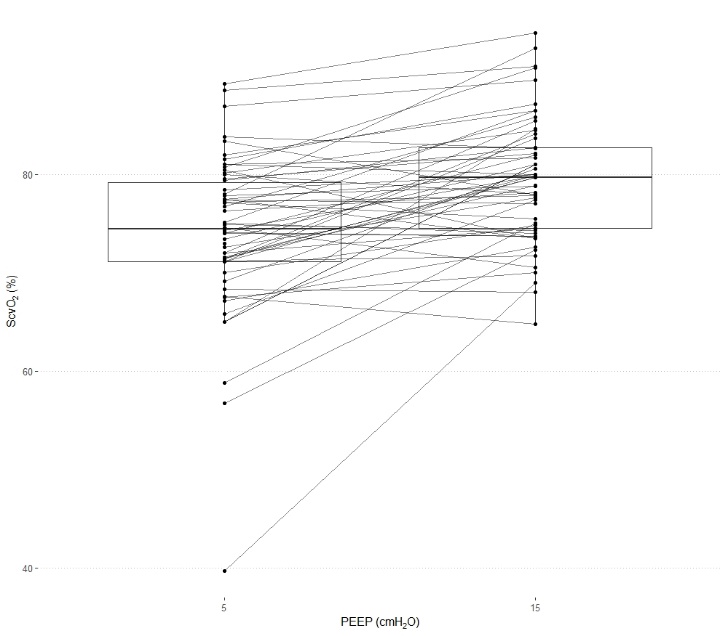 |
| 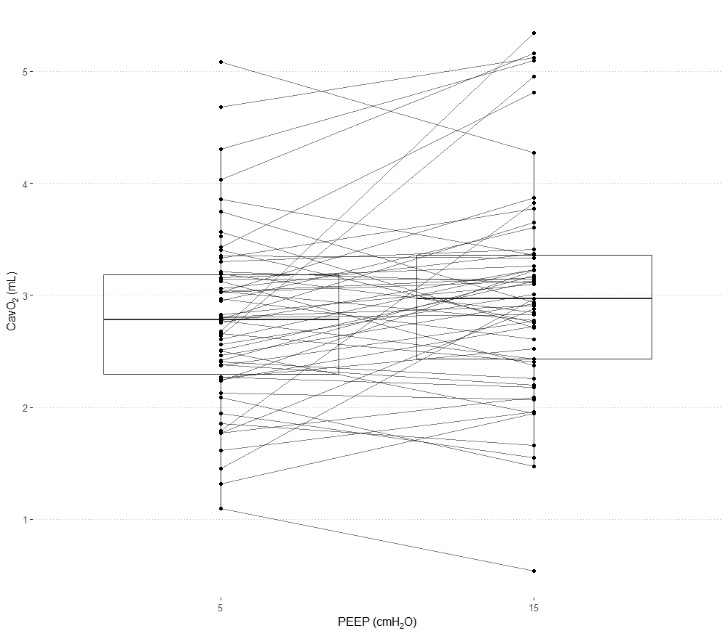 | 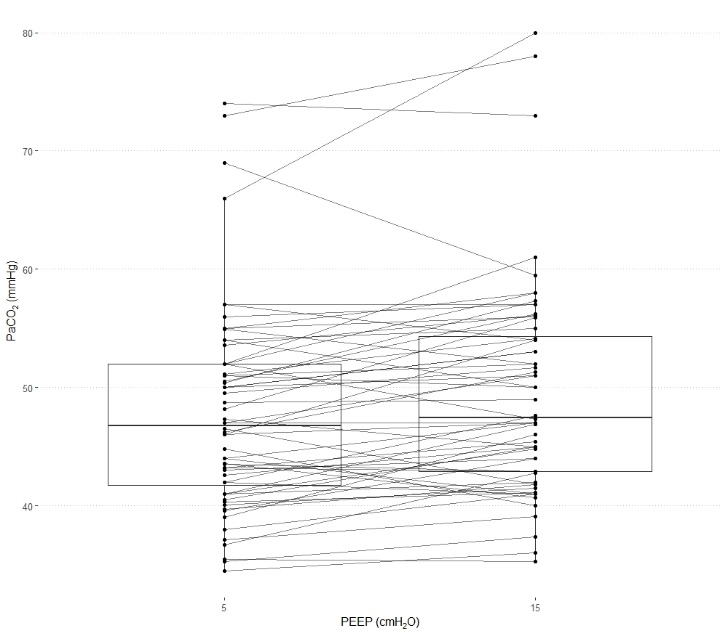 |
| 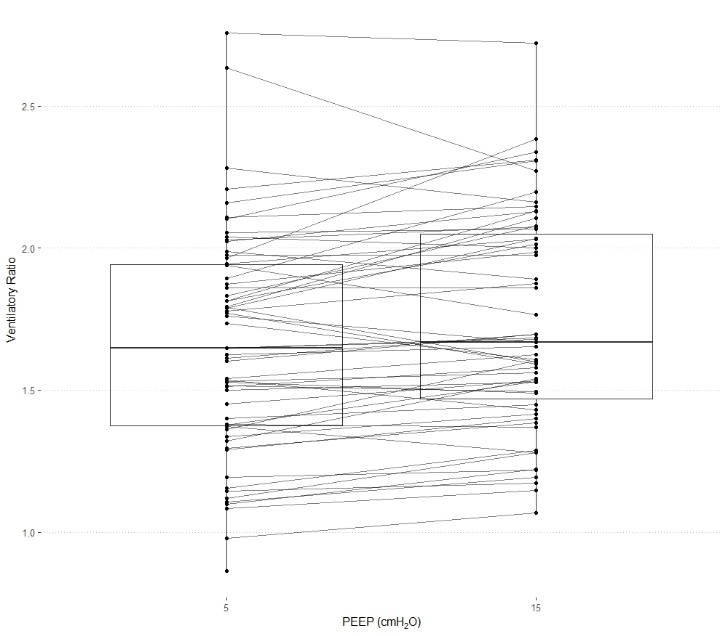 |  |

**Figure 3S.** Boxplot and two-point graphical representation showing each patient’s variable at 5 and 15 cmH_2_O of PEEP within the low respiratory system compliance group (Crs < 44 mL/cmH_2_O) and high respiratory system compliance group (Crs $\boldsymbol{\geq}$ 44 mL/cmH_2_O).

| 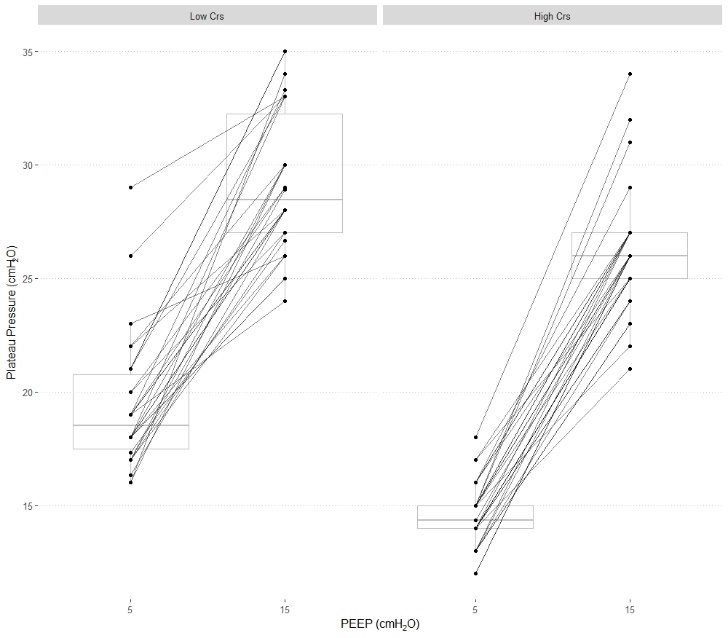 | 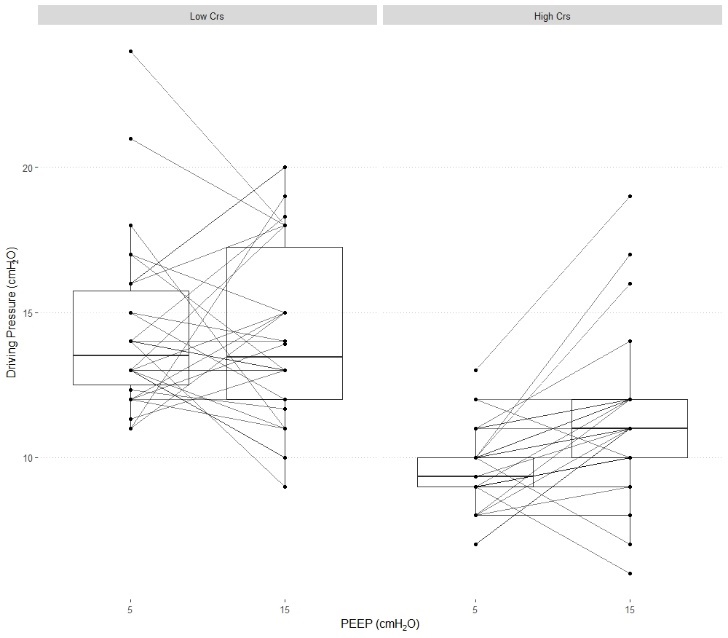 |
| --- | --- |
| 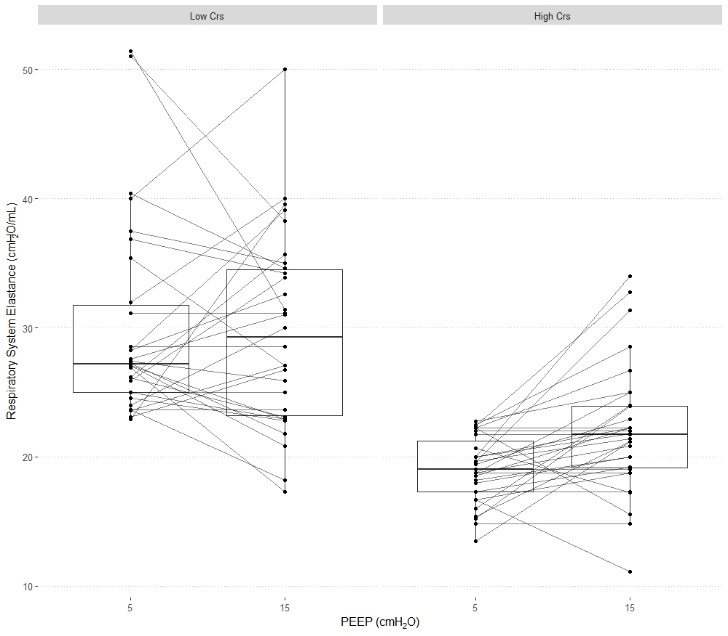 | 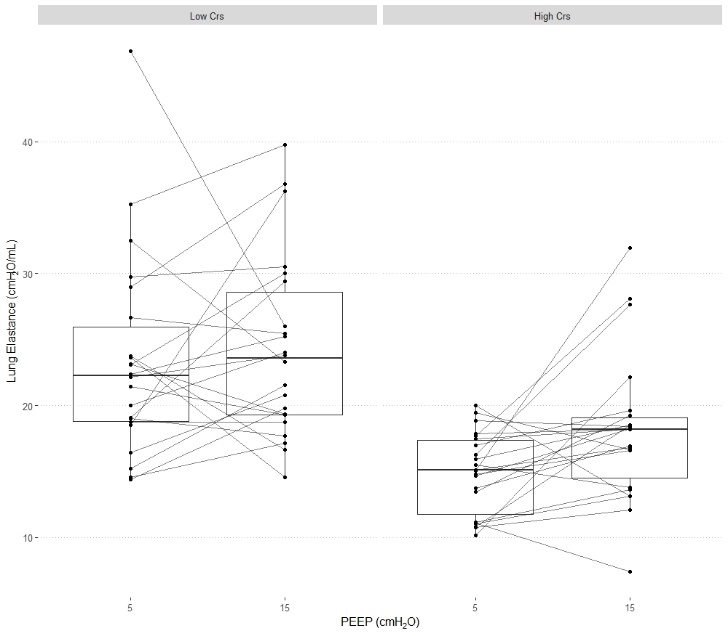 |
| 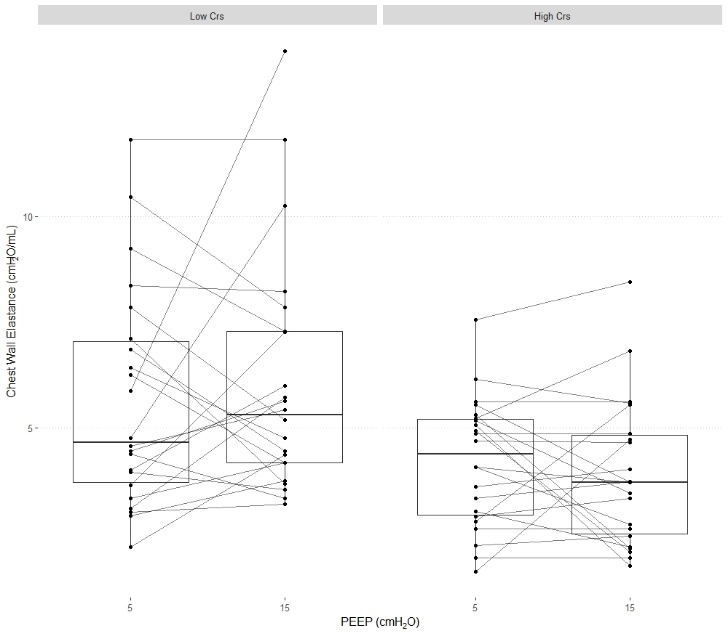 | 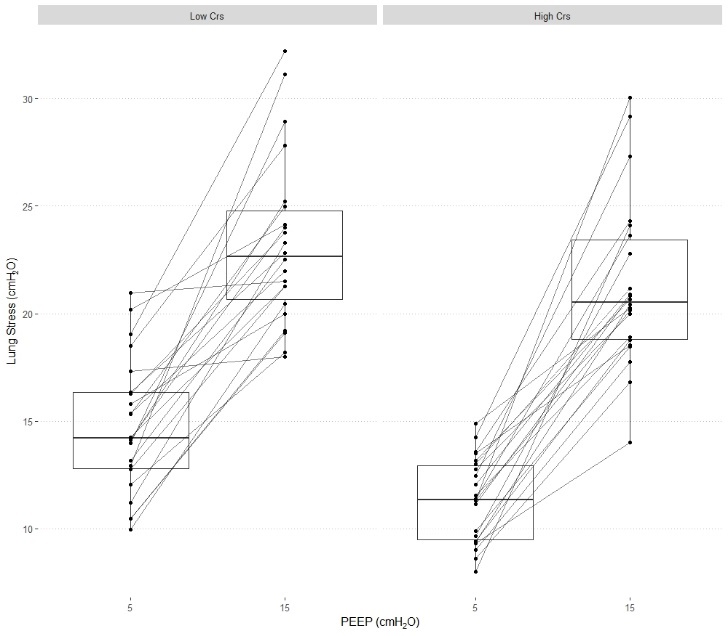 |
| 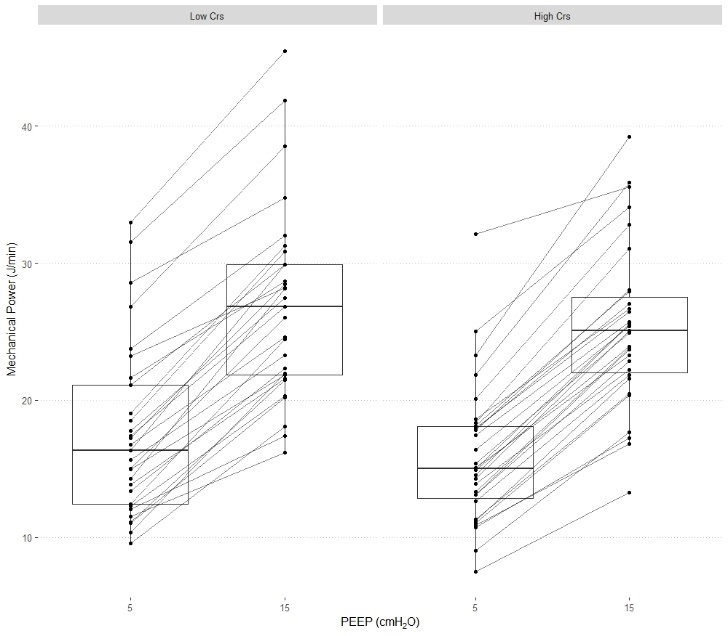 | 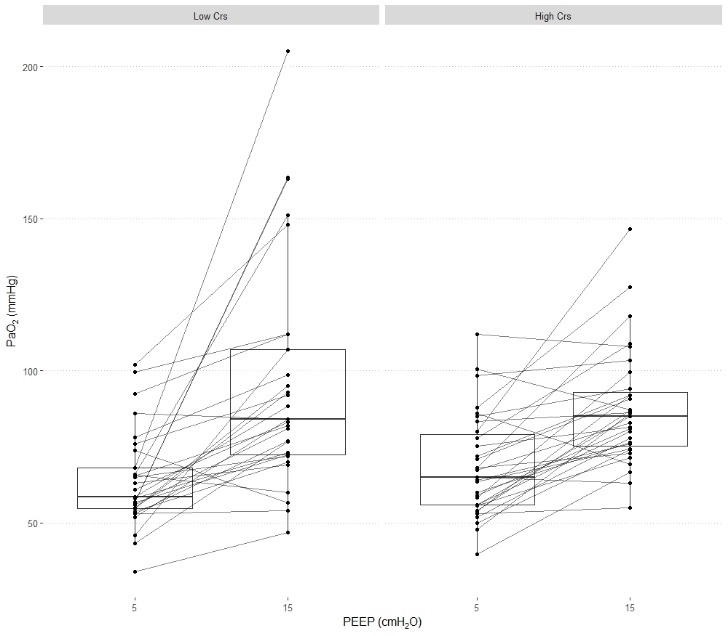 |
| 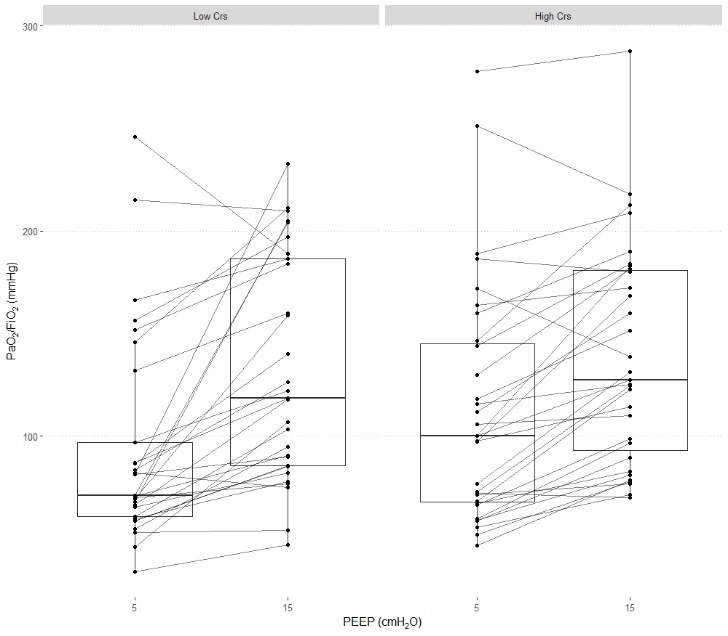 | 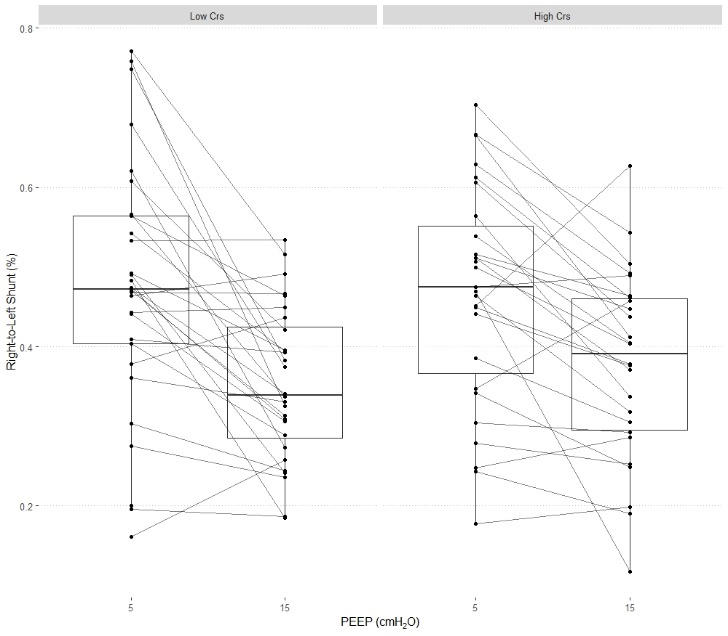 |
| 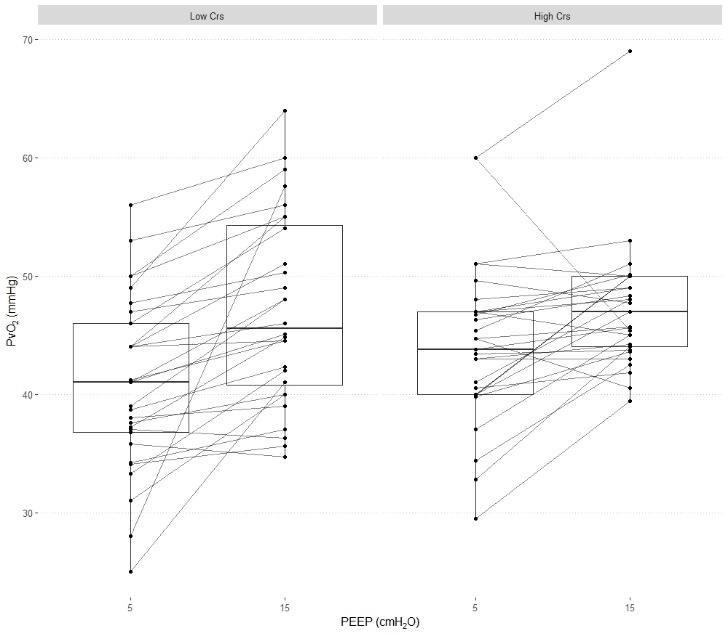 | 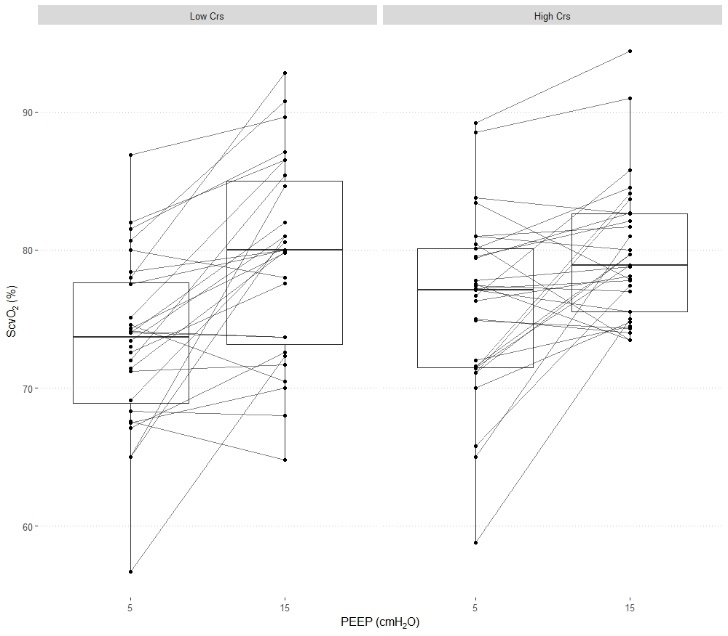 |
| 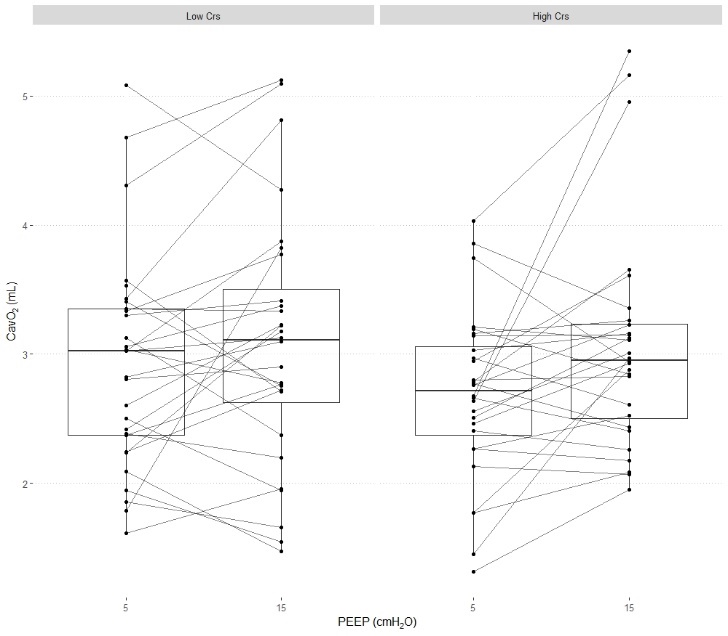 | 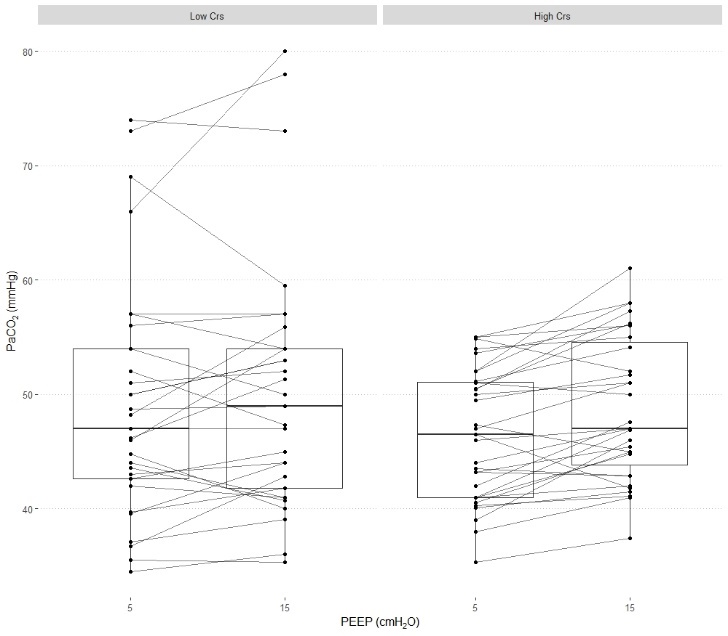 |
| 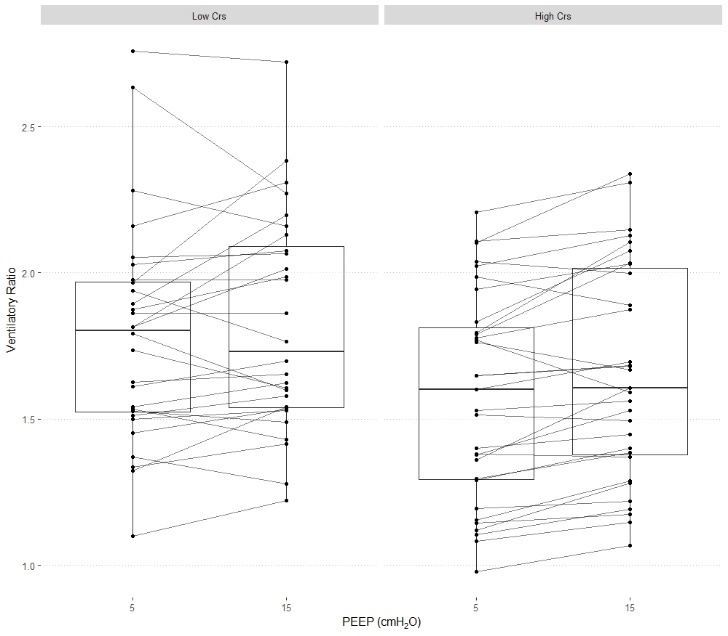 |  |

**Figure 4S.** Boxplot and two-point graphical representation showing each patient’s variable at 5 and 15 cmH_2_O of PEEP within the low PaO_2_/FiO_2_ group (PaO_2_/FiO_2_ < 81.8) and high PaO_2_/FiO_2_ group (PaO_2_/FiO_2_ $\boldsymbol{\geq}$ 81.8).

| 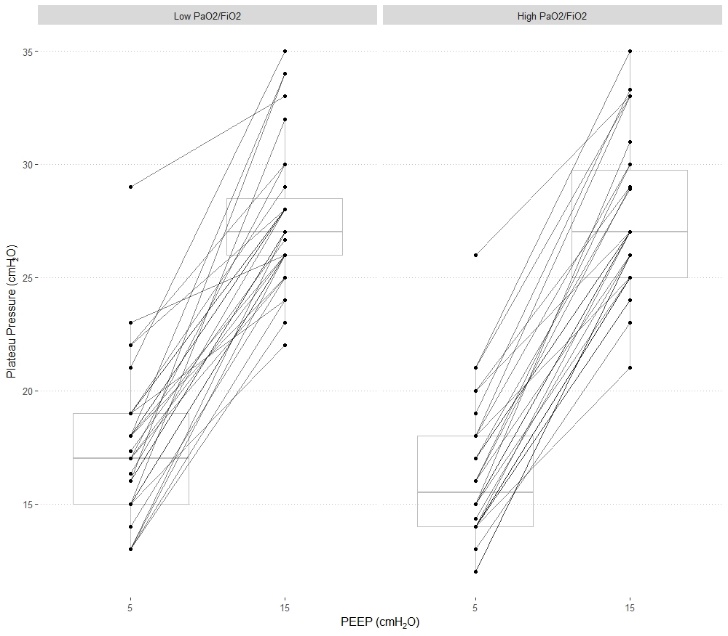 | 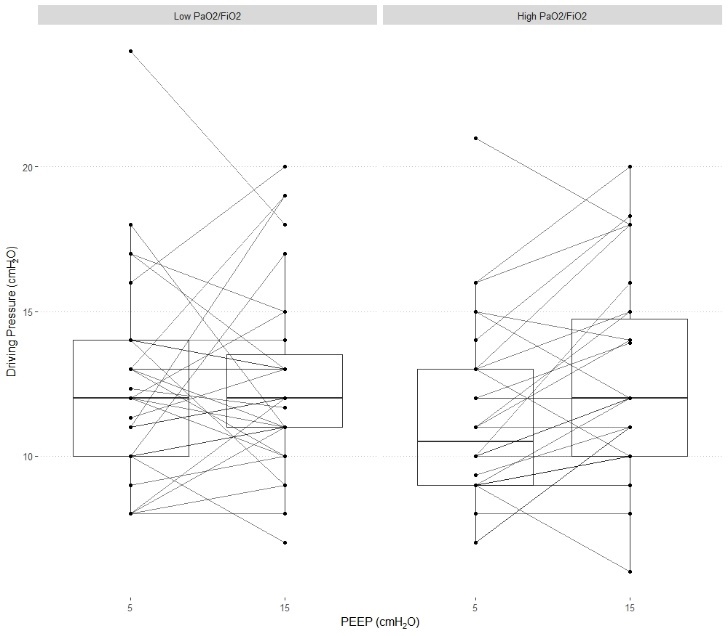 |
| --- | --- |
| 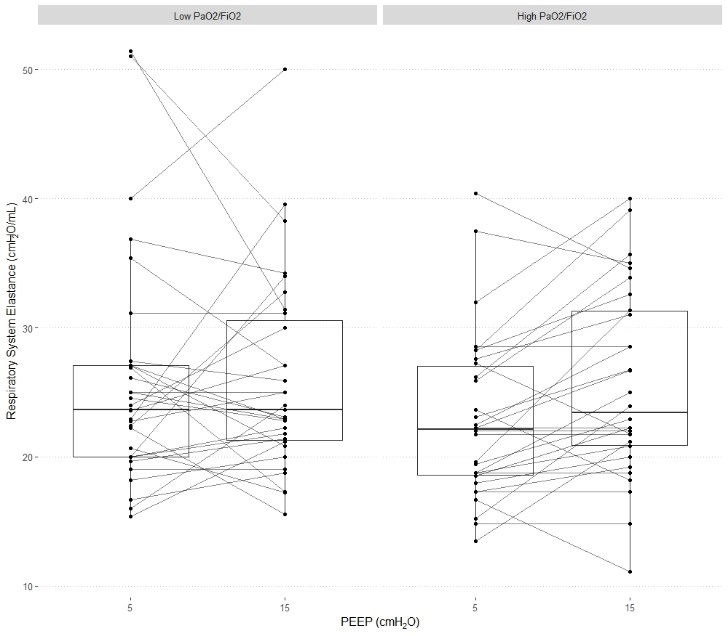 | 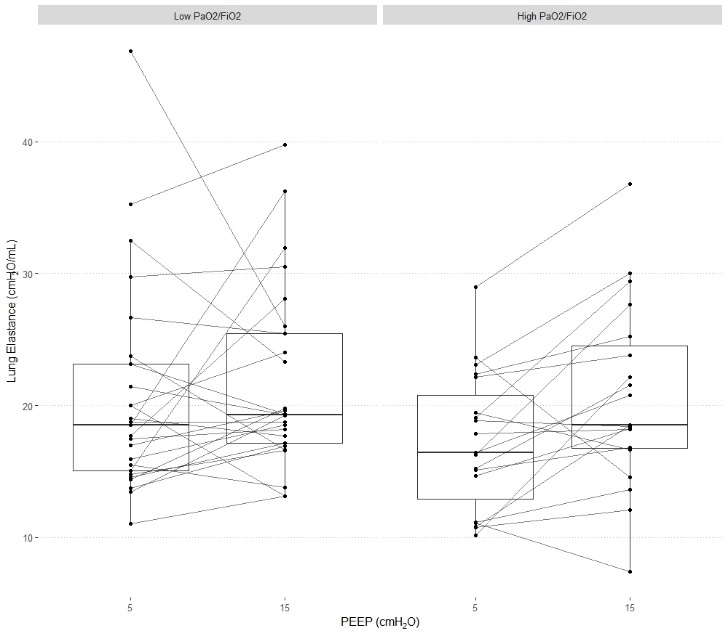 |
| 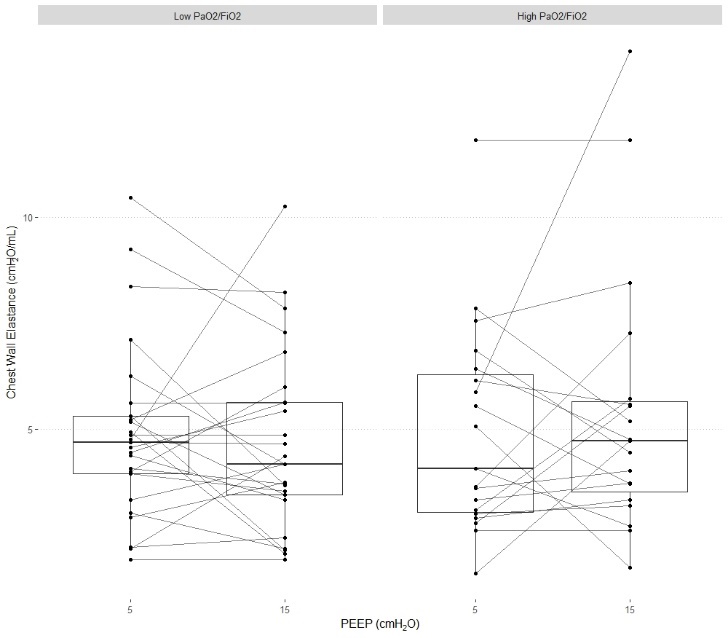 | 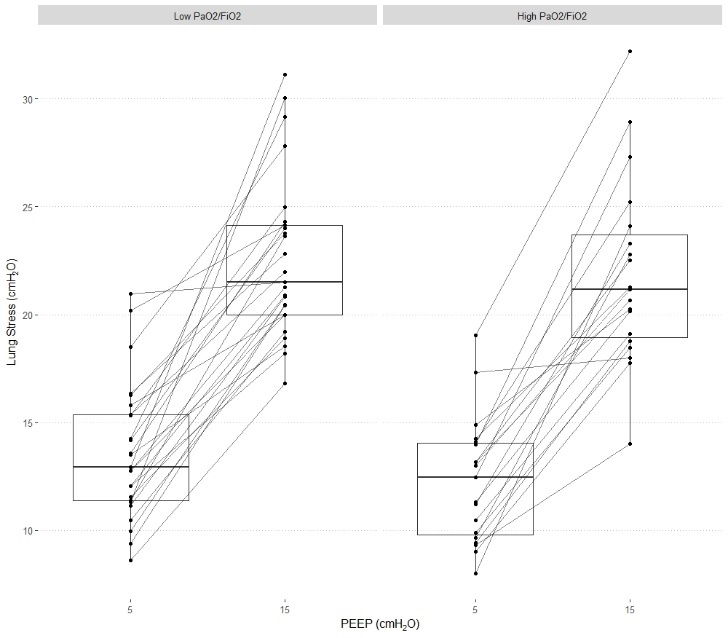 |
| 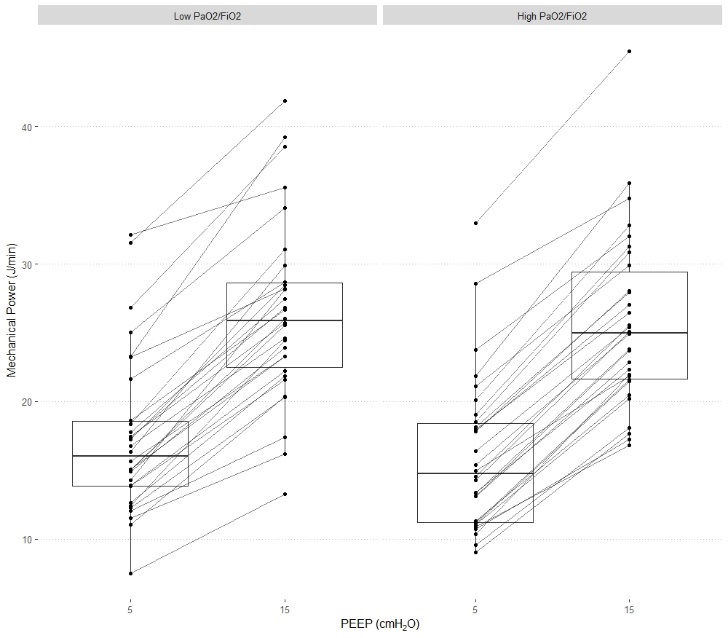 | 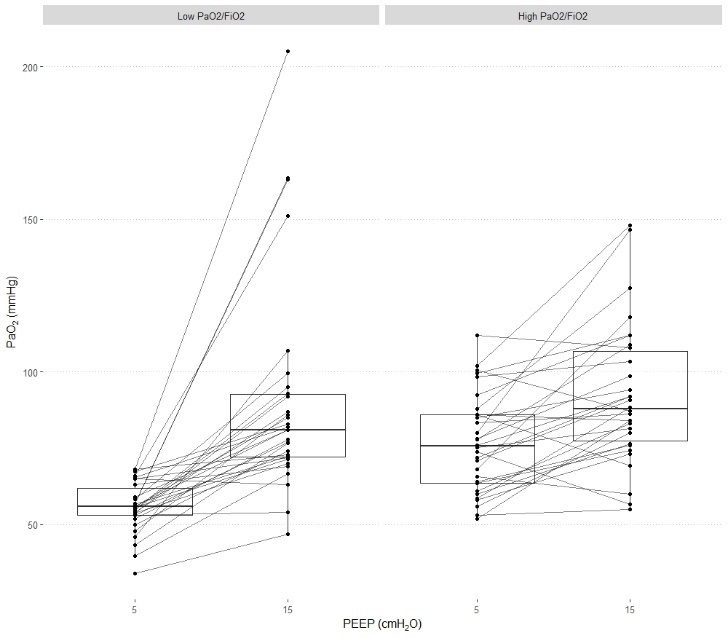 |
| 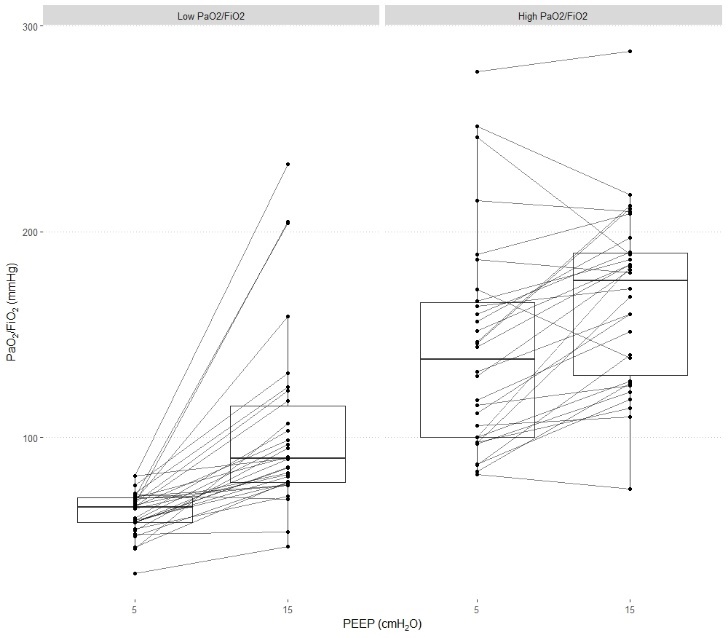 | 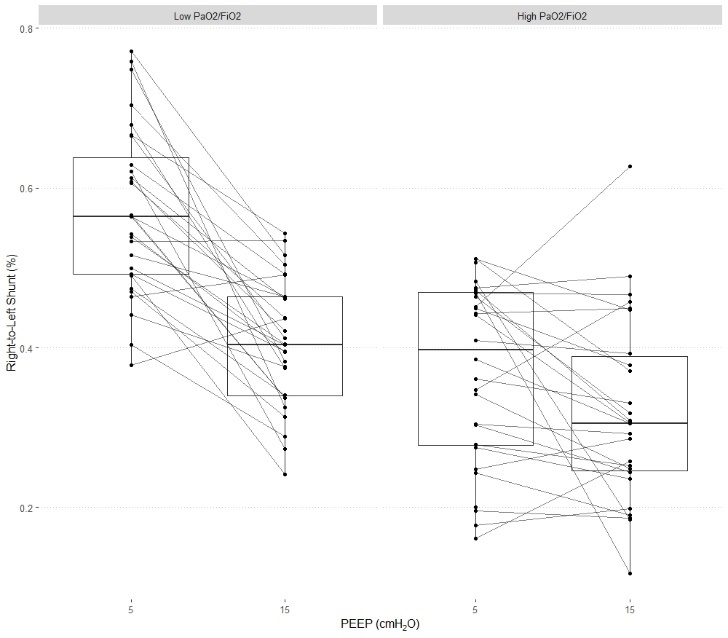 |
| 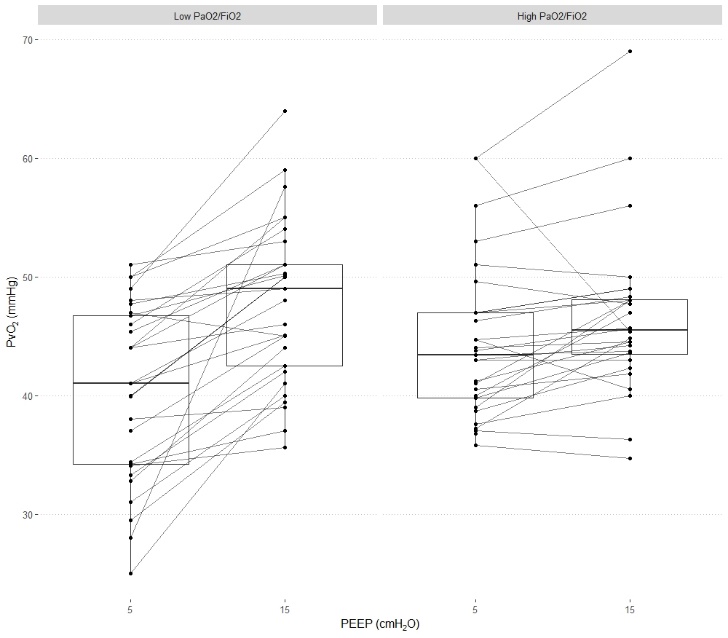 | 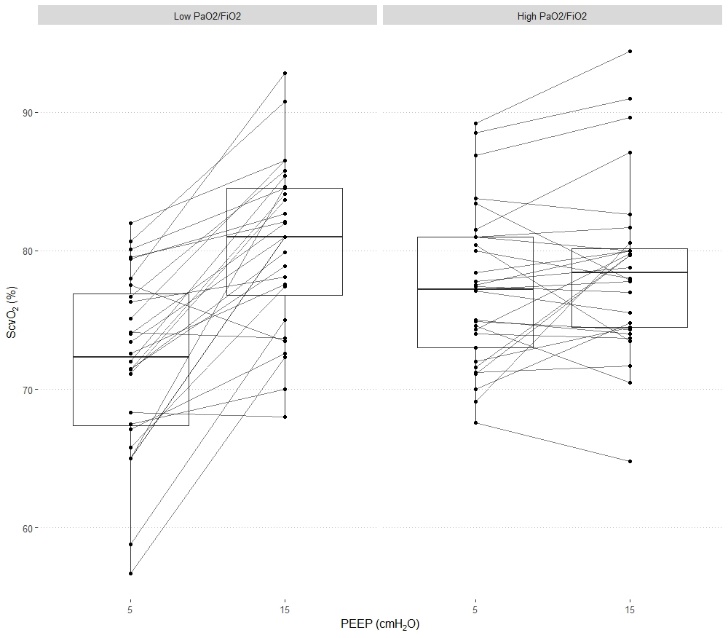 |
| 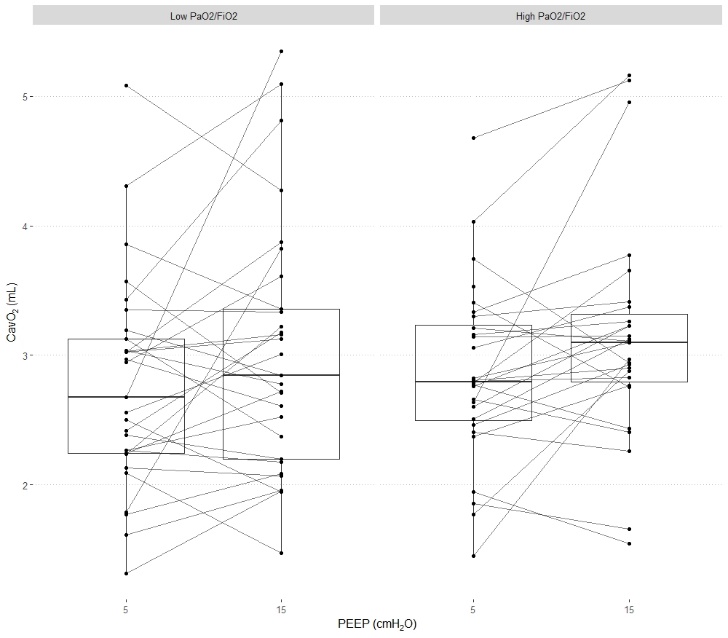 | 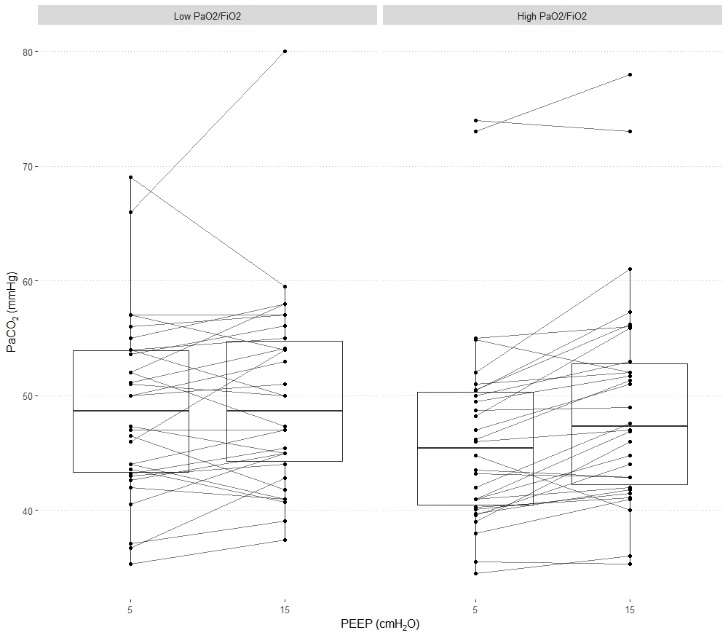 |
| 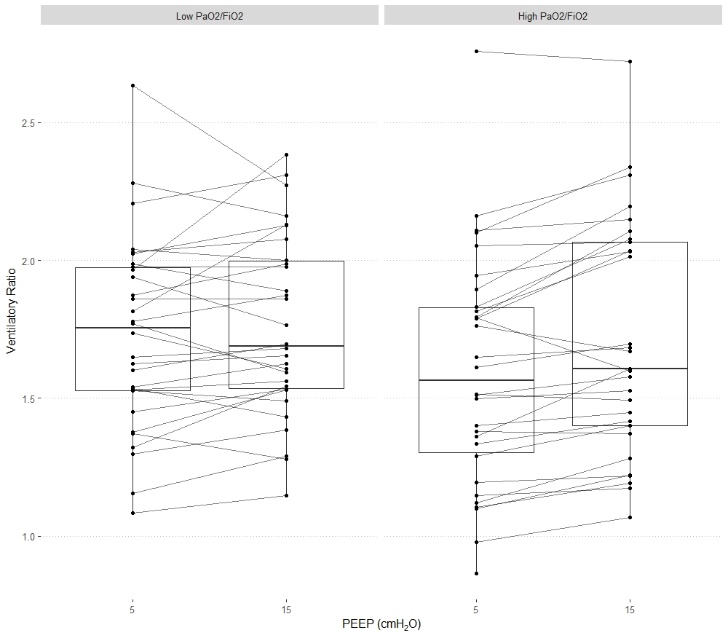 |  |

**Table 2S**. Respiratory mechanics, gas exchange and computed-tomography variables within high and low respiratory system compliance group. T-test or Wilcoxon-Mann-Whitney test were performed, as appropriate.

| Variables at PEEP 5 | Low C_rs_  < 44  *mL/cmH_2_O*  30 patients | High C_rs_  $\boldsymbol{\geq}$44  *mL/cmH_2_O*  31 patients | *p* |
| --- | --- | --- | --- |
| Age, *years* | 60 ± 9 | 61 ± 8 | *0.522* |
| Male sex, % (n) | 73 (22) | 98 (30) | ***0.026*** |
| Body Mass Index, *kg/m^2^* | 27 [26 – 32] | 28 [26 – 32] | *0.789* |
| SAPS II | 33 [27 – 35] | 32 [28 – 38] | *0.564* |
| Time between ETI and study day, *days* | 4 [2 – 7] | 3 [2 – 6] | *0.612* |
| Tidal volume/PBW, *mL/kg* | 7.4 [6.8 – 7.8] | 7.1 [6.8 – 7.5] | *0.471* |
| Respiratory rate, *bpm* | 18 [17 – 20] | 18 [16 – 20] | *0.617* |
| Minute ventilation, *L/min* | 9.1 ± 1.9 | 9.2 ± 1.6 | *0.905* |
| Plateau pressure, *cmH_2_O* | 18 [17 – 21] | 14 [14 – 15] | ***<0.001*** |
| Driving pressure, *cmH_2_O* | 13 [12 – 16] | 9 [9 – 10] | ***<0.001*** |
| Respiratory system compliance,  *mL/cmH_2_O* | 37 [31 – 40] | 52 [47 – 58] | ***<0.001*** |
| Lung stress, *cmH_2_O* | 15 ± 3 | 11 ± 2 | ***<0.001*** |
| Mechanical power, *J/min* | 16 [12 – 21] | 15 [13 – 18] | *0.429* |
| Mechanical Power_Compliance_rs_ , *J/min(mL/cmH_2_O)* | 0.47 [0.36 – 0.59] | 0.28 [0.24 – 0.35] | ***<0.001*** |
| Arterial pH | 7.39 ± 0.07 | 7.38 ± 0.07 | *0.762* |
| PaO_2_, *mmHg* | 59 [55 – 68] | 65 [56 – 79] | *0.284* |
| PaO_2_/FiO_2_, *mmHg* | 71 [61 – 97] | 100 [68 – 145] | *0.126* |
| Right-to-left shunt, *%* | 47 ± 16 | 46 ± 14 | *0.780* |
| PvO_2_, *mmHg* | 40 ± 7 | 44 ± 7 | *0.090* |
| ScvO_2_, *%* | 76 ± 7 | 78 ± 6 | *0.157* |
| C_a-v_O_2_, *mL* | 2.9 ± 0.8 | 2.6 ± 0.7 | *0.151* |
| PaCO_2_, *mmHg* | 47 [40 – 56] | 47 [35 – 56] | *0.534* |
| Ventilatory ratio | 1.75 ± 0.41 | 1.58 ± 0.36 | *0.084* |
| Estimated physiological dead space | 0.51 ± 0.12 | 0.46 ± 0.14 | *0.147* |
| Outcome, *% (n)*  Dead  Alive | 47 (14)  53 (16) | 45 (14)  55 (17) | *0.999* |
| Total lung weight, *g* | 1207 [937 – 1478] | 1546 [1229 – 1789] | ***0.029*** |
| Total gas volume, *mL* | 1098 [644 – 1547] | 1998 [1435 – 2574] | ***<0.001*** |
| Total lung volume, *mL* | 2121 [1865 – 2952] | 3585 [3035 – 4229] | ***<0.001*** |
| Over inflated tissue, *g* | 3 [1 – 6] | 7 [2 – 16] | ***0.047*** |
| Well inflated tissue, *g* | 329 ± 167 | 539 ± 222 | ***<0.001*** |
| Poorly inflated tissue, *g* | 429 ± 243 | 451 ± 244 | *0.742* |
| Non inflated tissue, *g* | 261 [129 – 567] | 387 [138 – 598] | *0.446* |
| Over inflated tissue, *%* | 1.5 [0.3 – 3.5] | 4.5 [0.7 – 8.4] | *0.284* |
| Well inflated tissue, *%* | 54.4 ± 14.1 | 63.7 ± 13.0 | ***0.015*** |
| Poorly inflated tissue, *%* | 23.3 [19.9 – 33.7] | 18.3 [12.8 – 22.5] | ***0.007*** |
| Non inflated tissue, *%* | 10.1 [4.7 – 21.0] | 10.8 [2.7 – 17.7] | *0.645* |

SAPS II: Simplified Acute Physiology Score; C_a-v_O_2_: arterial-venous oxygen content difference; Compliance_rs_ and C_rs_: respiratory system compliance; ScvO_2_ central oxygen venous saturation; PvO_2_ mixed venous oxygen tension

**Table 3S**. Respiratory mechanics, gas exchange and computed-tomography variables within high and low PaO_2_/FiO_2_ group. T-test or Wilcoxon-Mann-Whitney test were performed, as appropriate.

| Variables at PEEP 5 | Low  PaO_2_/FiO_2_ < 81.8  31 patients | High  PaO_2_/FiO_2_ $\boldsymbol{\geq}$ 81.8  30 patients | *p* |
| --- | --- | --- | --- |
| Age, *years* | 60 ± 8 | 61 ± 9 | *0.697* |
| Male sex, *% (n)* | 77 (24) | 93 (28) | *0.164* |
| Body Mass Index, *kg/m^2^* | 28 [26 – 34] | 28 [26 – 29] | *0.352* |
| SAPS II | 34 [30 – 37] | 31 [28 – 36] | *0.158* |
| Time between ETI and study day, *days* | 3 [2 – 4] | 5 [3 – 8] | ***0.019*** |
| Tidal volume/PBW, mL/kg | 7.4 [6.8 – 7.8] | 6.7 [7.1 – 7.7] | *0.526* |
| Respiratory rate, bpm | 18 [16 – 20] | 18 [17 – 20] | *0.676* |
| Minute ventilation, *L/min* | 9.1 ± 1.7 | 9.2 ± 1.9 | *0.857* |
| Plateau pressure, *cmH_2_O* | 17 [15 – 19] | 15 [14 – 18] | *0.126* |
| Driving pressure, *cmH_2_O* | 12 [10 – 14] | 10 [9 – 13] | *0.126* |
| Respiratory system compliance,  *mL/cmH_2_O* | 42 ± 11 | 47 ± 12 | *0.121* |
| Lung stress, *cmH_2_O* | 14 ± 3 | 12 ± 3 | *0.160* |
| Mechanical power, *J/min* | 16 [14 – 19] | 15 [11 – 18] | *0.333* |
| Mechanical Power_Compliance_rs_, *J/min/(mL/cmH_2_O)* | 0.40 [0.30 – 0.54] | 0.31 [0.25 – 0.40] | ***<0.001*** |
| Arterial pH | 7.39 ± 0.07 | 7.38 ± 0.07 | *0.885* |
| PaO_2_, *mmHg* | 56 ± 8 | 77 ± 16 | ***<0.001*** |
| PaO_2_/FiO_2_, *mmHg* | 66 [59 – 71] | 138 [100 – 166] | ***<0.001*** |
| Right-to-left shunt, *%* | 0.56 [0.49 – 0.63] | 0.38 [0.28 – 0.47] | ***<0.001*** |
| PvO_2_, *mmHg* | 41 [34 – 47] | 43 [40 – 47] | *0.135* |
| ScvO_2_, *%* | 77 [71 – 81] | 78 [74 – 81] | *0.147* |
| C_a-v_O_2_, *mL* | 2.8 ± 0.8 | 2.8 ± 0.7 | *0.880* |
| PaCO_2_, *mmHg* | 49 [43 – 54] | 45 [40 – 50] | *0.105* |
| Ventilatory ratio | 1.73 ± 0.35 | 1.63 ± 0.41 | *0.175* |
| Estimated physiological dead space | 0.50 ± 0.10 | 0.46 ± 0.16 | *0.271* |
| Outcome, *% (n)*  Dead  Alive | 58 (18)  42 (13) | 33 (10)  67 (20) | *0.093* |
| Total lung weight, *g* | 1343 [1047 - 1632] | 1365 [932 – 1719] | *0.668* |
| Total gas volume, *mL* | 1197 [724 – 1930] | 1534 [1125 – 2290] | *0.105* |
| Total lung volume, *mL* | 2410 [1935 – 3506] | 3227 [2582 – 3714] | *0.106* |
| Over inflated tissue, *g* | 3 [1 – 8] | 5 [1 – 15] | *0.145* |
| Well inflated tissue, *g* | 384 ± 214 | 471 ± 222 | *0.147* |
| Poorly inflated tissue, *g* | 321 [171 – 511] | 252 [110 – 753] | *0.807* |
| Not inflated tissue, *g* | 347 ± 239 | 428 ± 402 | *0.369* |
| Over inflated tissue, % | 1.3 [0.4 – 6.2] | 3.3 [0.6 – 7.1] | *0.381* |
| Well inflated tissue, % | 55.5 ± 13.7 | 62.0 ± 14.3 | *0.089* |
| Poorly inflated tissue, *%* | 27.0 ± 12.9 | 19.0 ± 7.7 | ***0.007*** |
| Not inflated tissue, *%* | 10.7 [6.0 – 18.7] | 9.6 [2.5 – 21.1] | *0.757* |

SAPS II: Simplified Acute Physiology Score; C_a-v_O_2_: arterial-venous oxygen content difference; Compliance_rs_: respiratory system compliance; ScvO_2_ central oxygen venous saturation; PvO_2_ mixed venous oxygen tension.

**Figure 5S**. Linear regression between the change from 5 to 15 cmH_2_O of PEEP of each variables and respiratory system compliance.

| 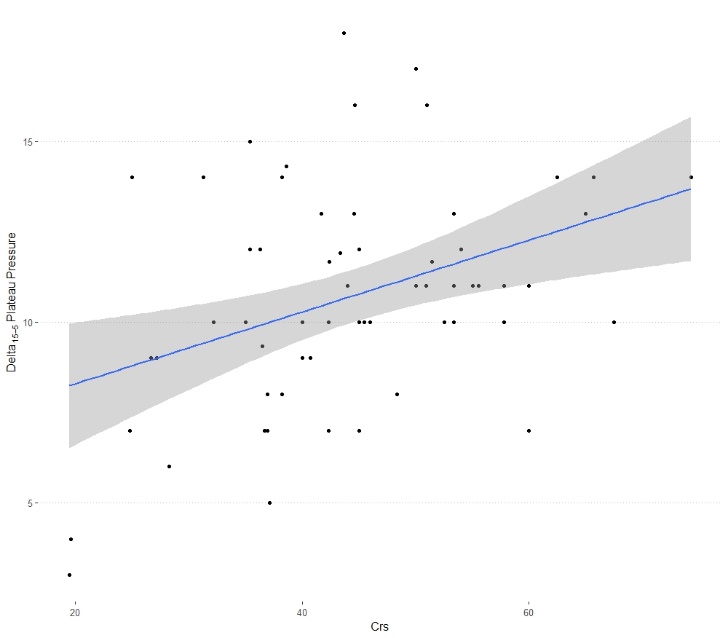 | **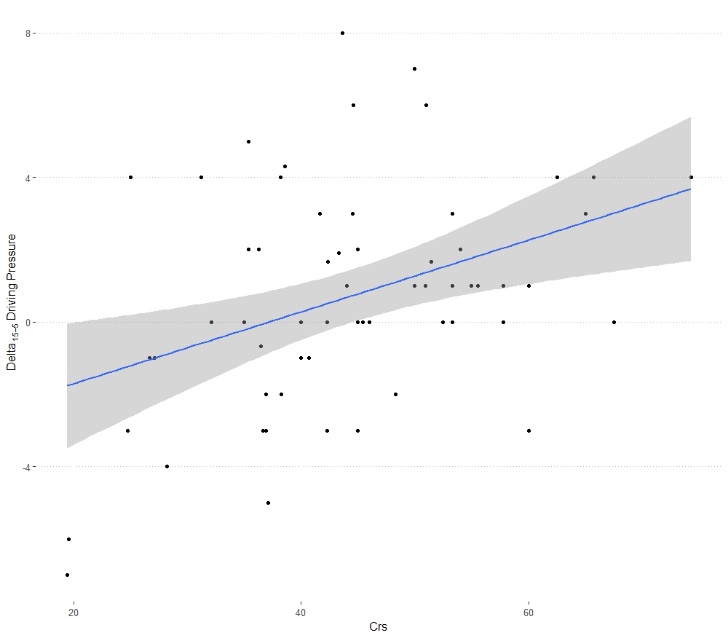** |
| --- | --- |
| 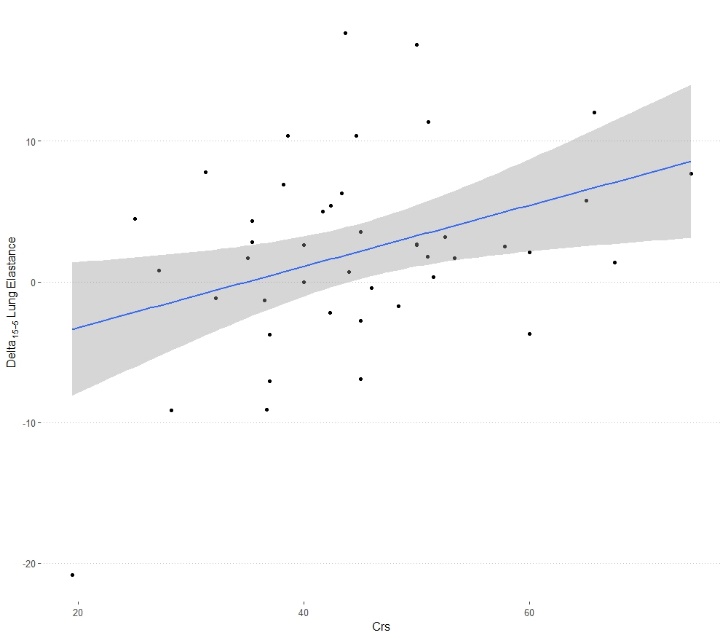 | 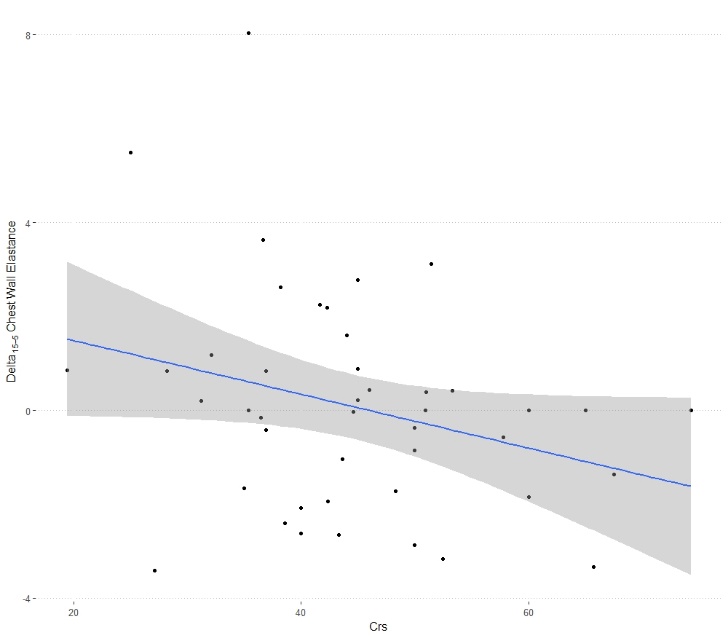 |
| 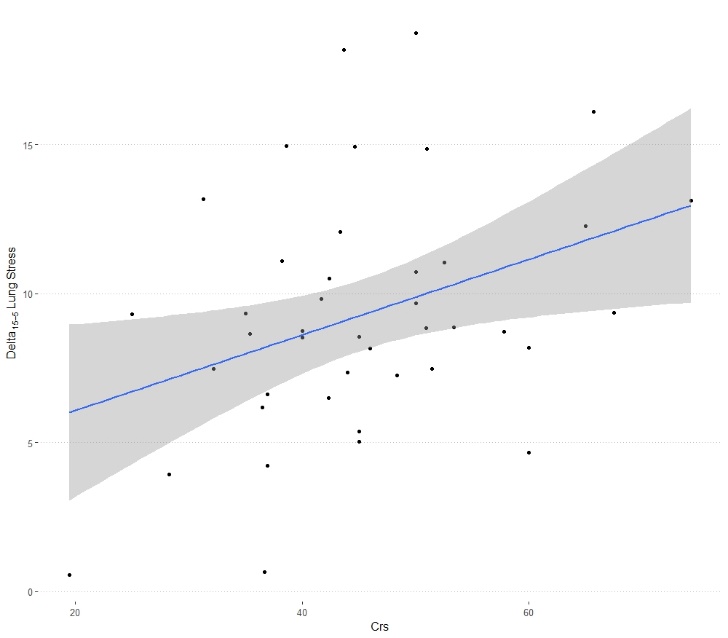 | 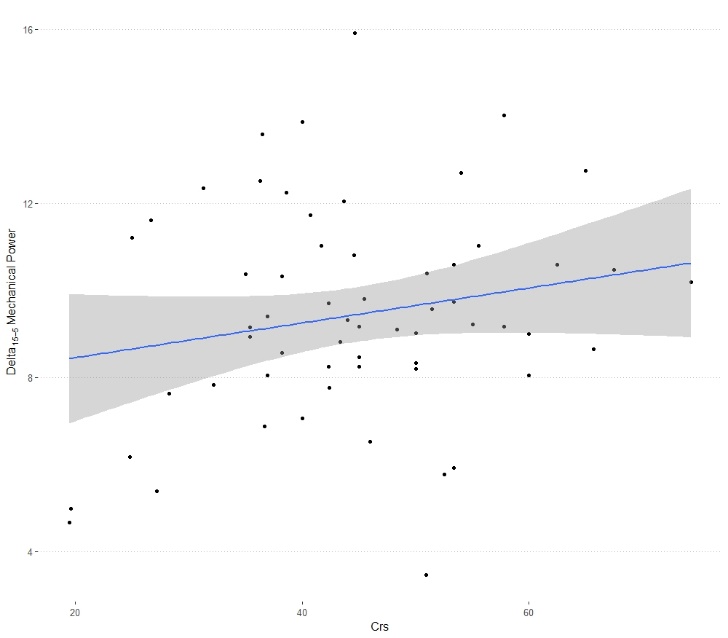 |
| 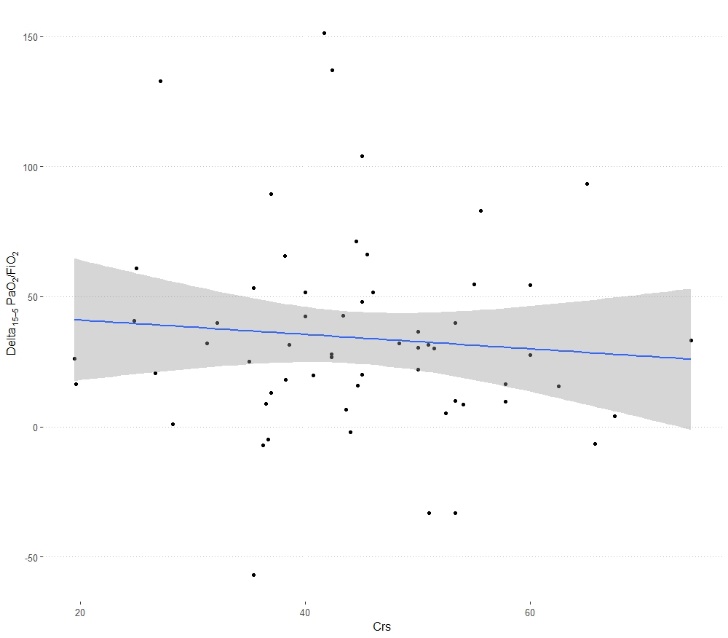 | ***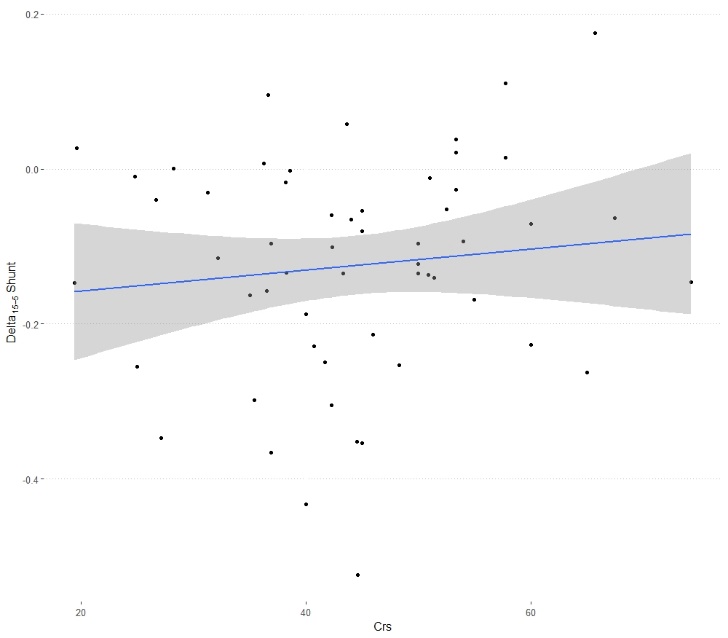*** |
| 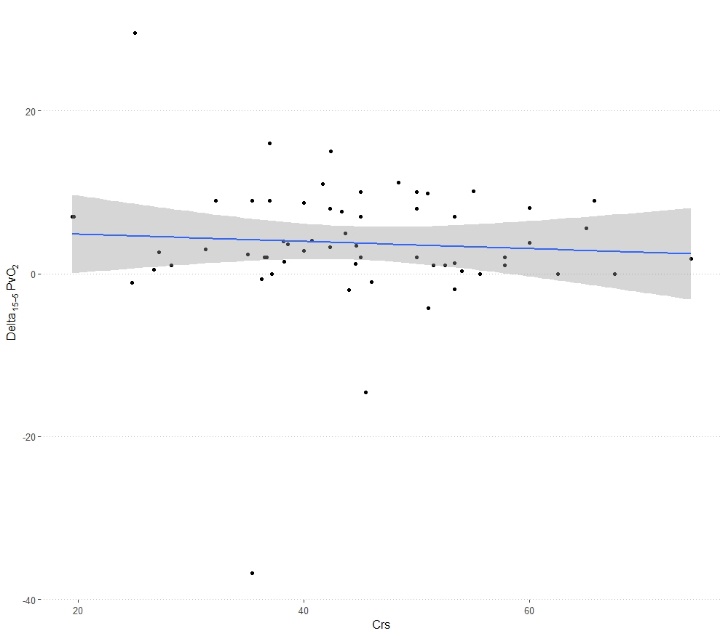 | 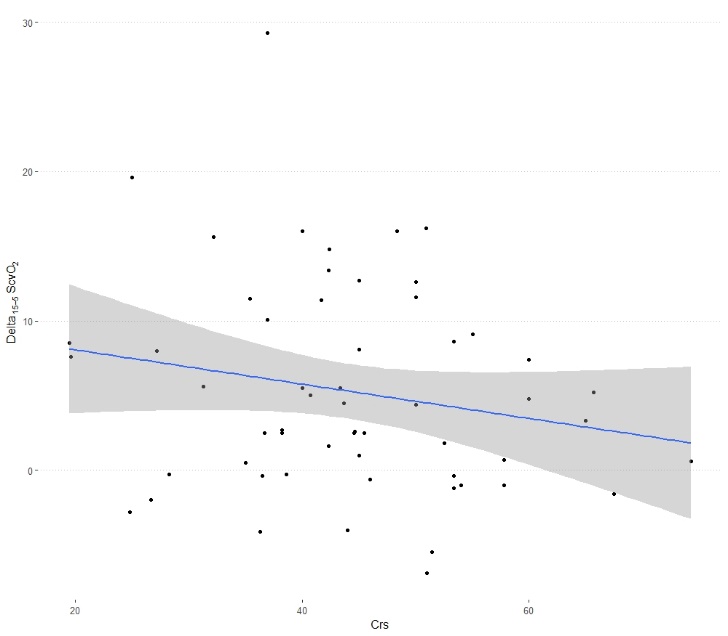 |
| *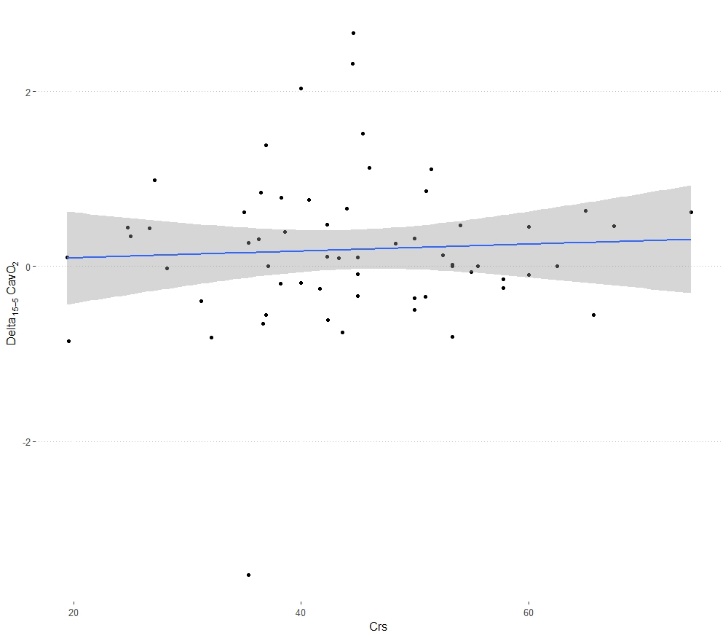* | 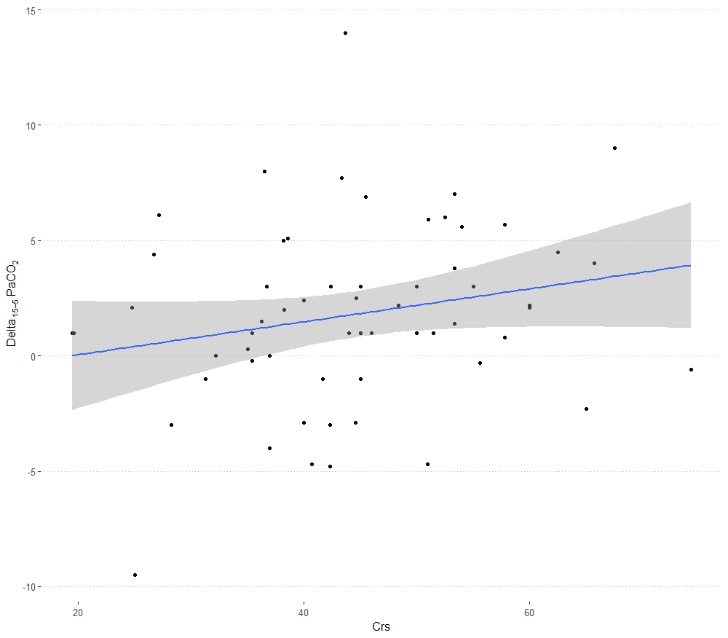 |
| *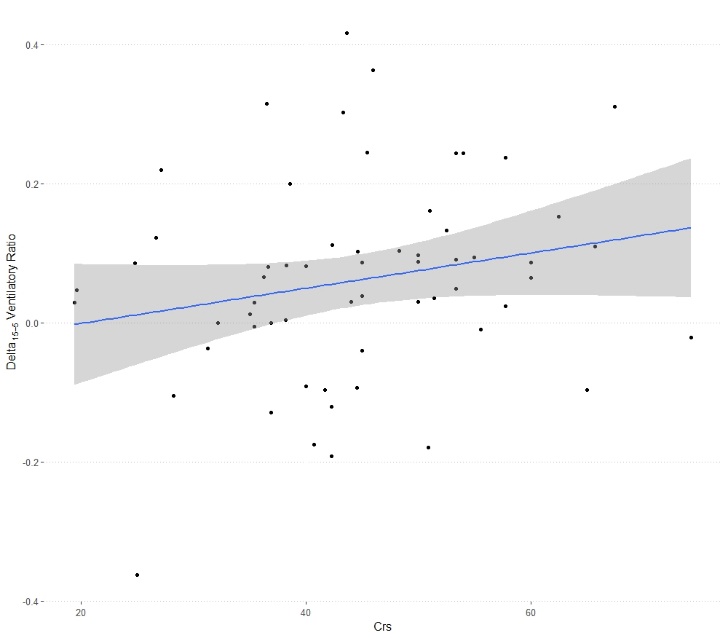* | *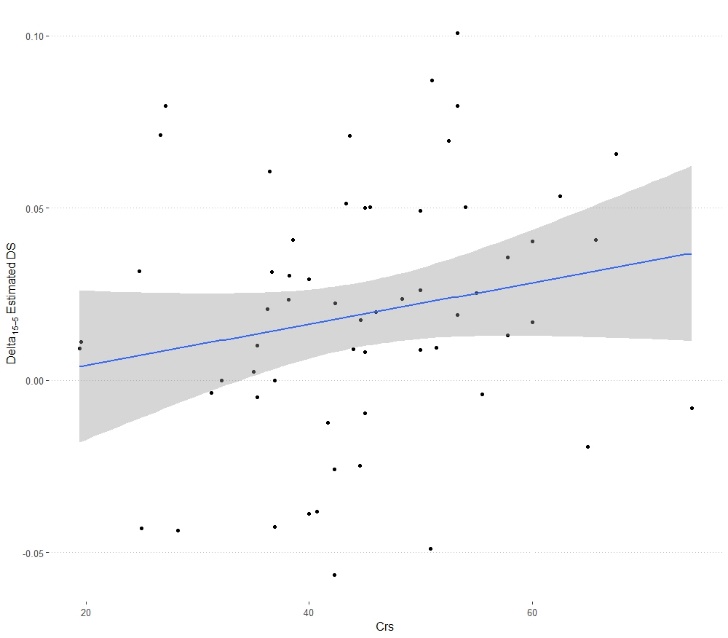* |

| Variables | R^2^ |
| --- | --- |
| Δ_15-5_ Plateau pressure, *cmH_2_O* | 0.133 |
| Δ_15-5_ Driving pressure, *cmH_2_O* | 0.133 |
| Δ_15-5_ Lung elastance, *cmH_2_O/L* | 0.115 |
| Δ_15-5_ Chest wall elastance, *cmH_2_O/L* | 0.061 |
| Δ_15-5_ Lung stress, *cmH_2_O* | 0.109 |
| Δ_15-5_ Mechanical power, *J/min* | 0.021 |
| Δ_15-5_ PaO_2_, *mmHg* | 0.009 |
| Δ_15-5_ PaO_2_/FiO_2_, *mmHg* | 0.007 |
| Δ_15-5_ Right-to-left shunt, *%* | 0.013 |
| Δ_15-5_ PvO_2_, *mmHg* | 0.004 |
| Δ_15-5_ ScvO_2_, *%* | 0.020 |
| Δ_15-5_ CavO_2_, *mL* | 0.003 |
| Δ_15-5_ PaCO_2_, *mmHg* | 0.030 |
| Δ_15-5_ Ventilatory ratio | 0.029 |
| Δ_15-5_ Estimated physiological dead space | 0.021 |

**Figure 6S**. Linear regression between the change from 5 to 15 cmH_2_O of PEEP of each variables and PaO_2_/FiO_2_.

| 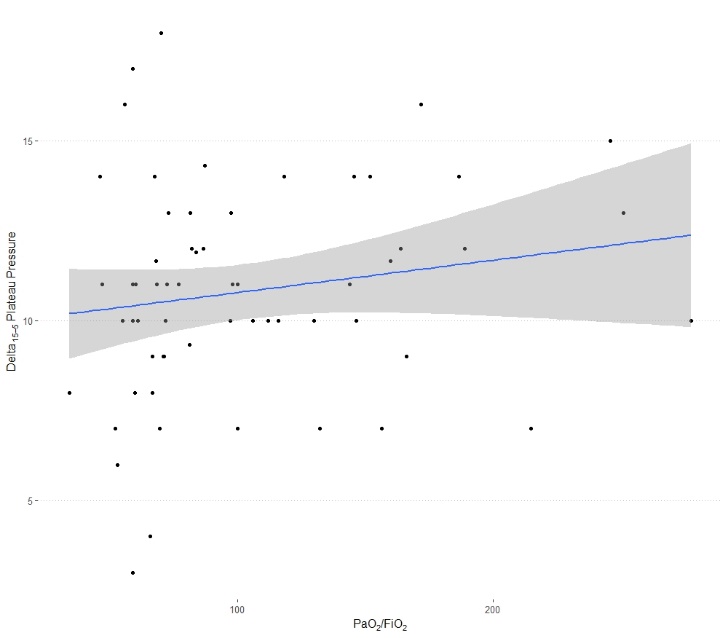 | *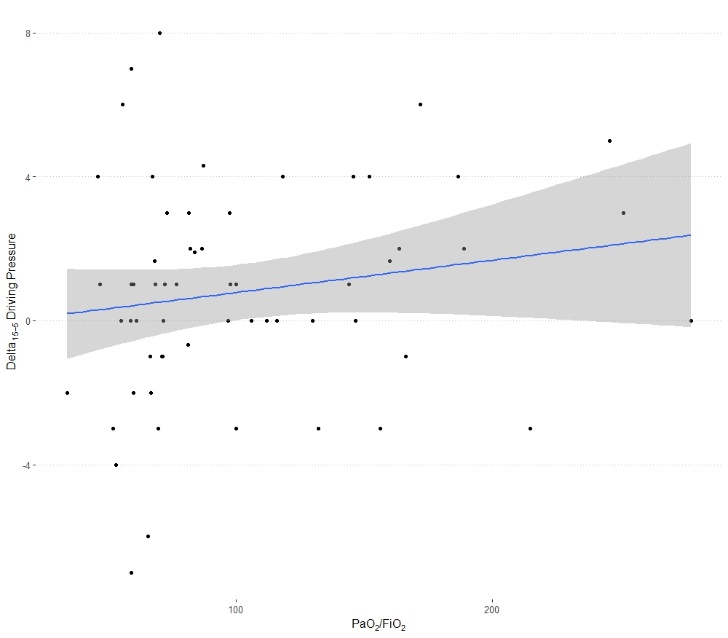* |
| --- | --- |
| 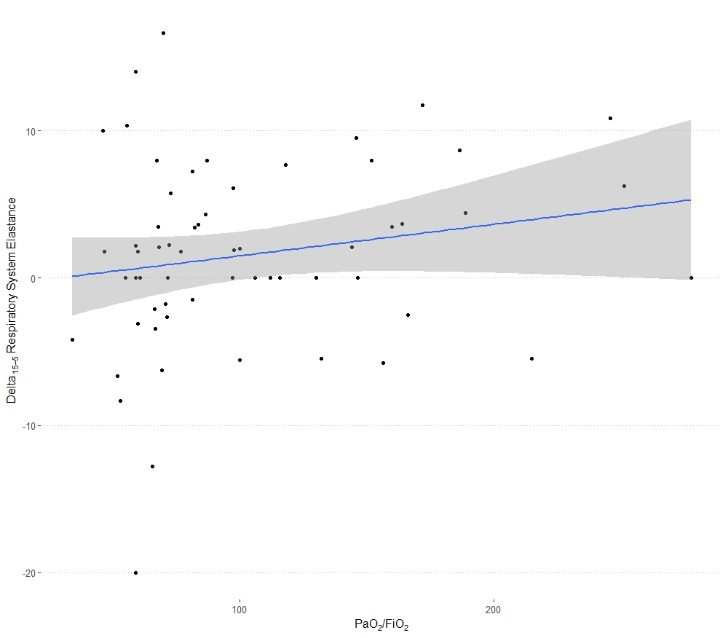 | 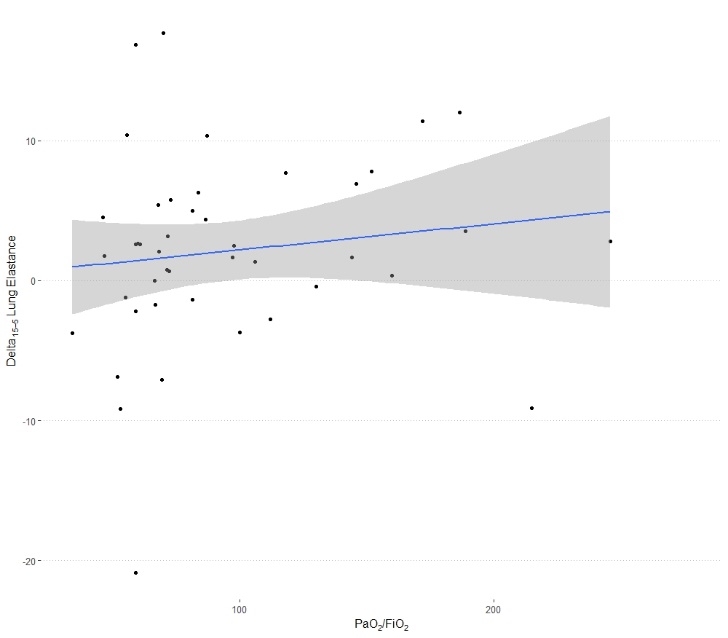 |
| 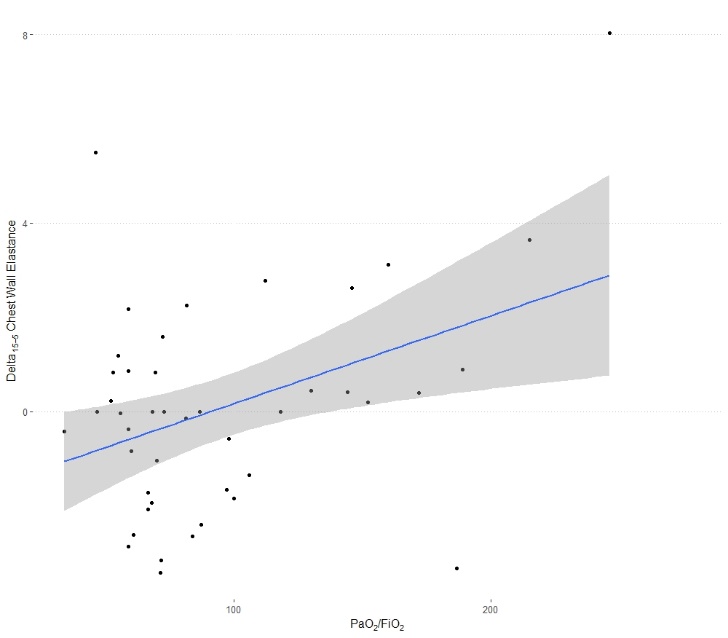 | 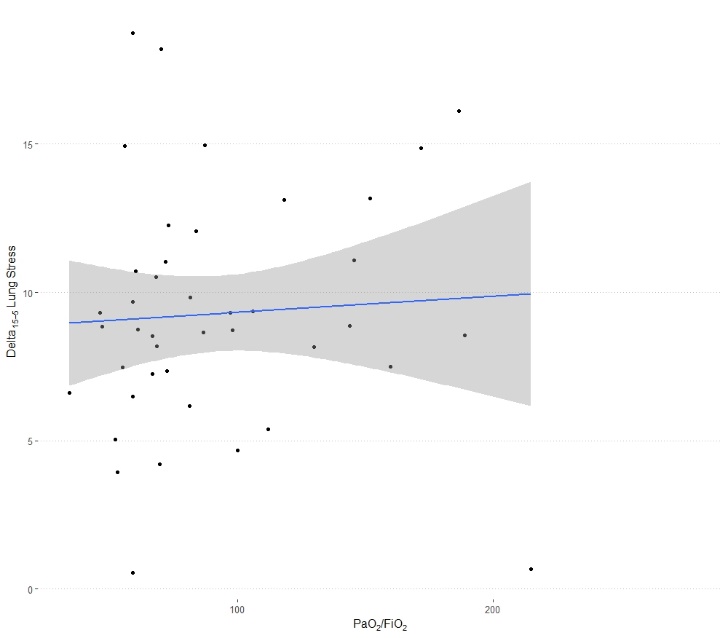 |
| 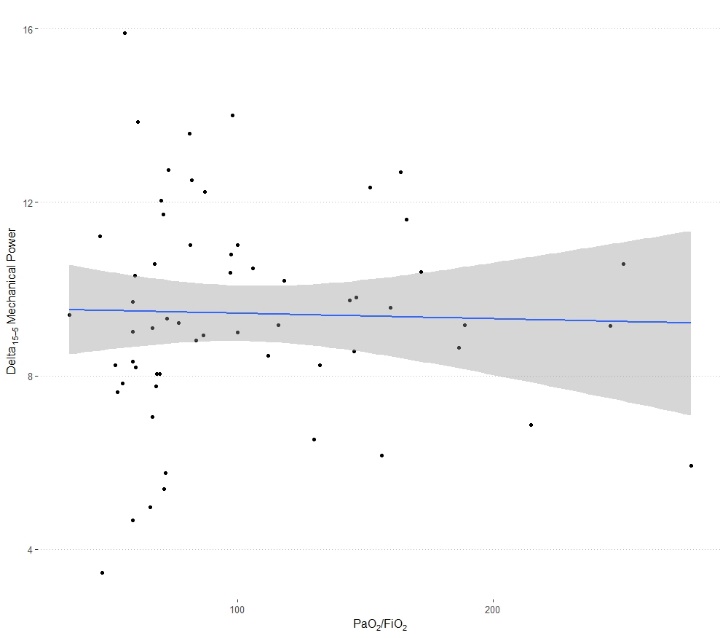 | 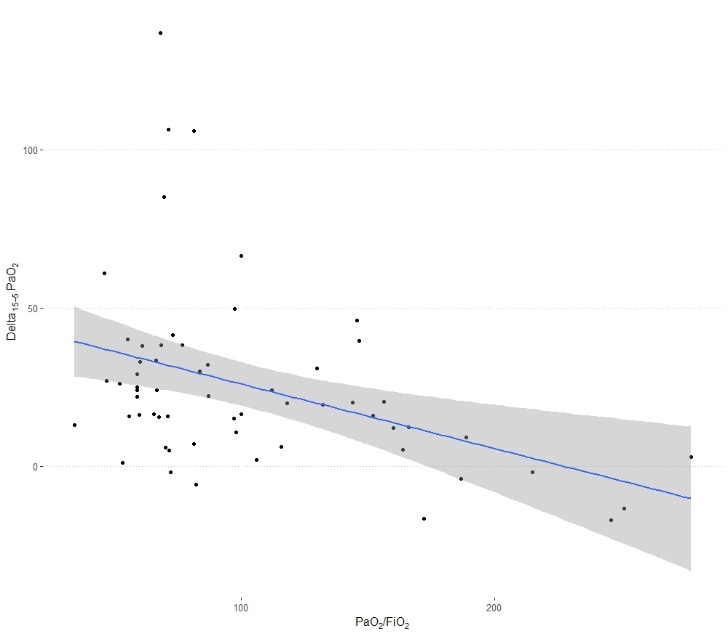 |
| 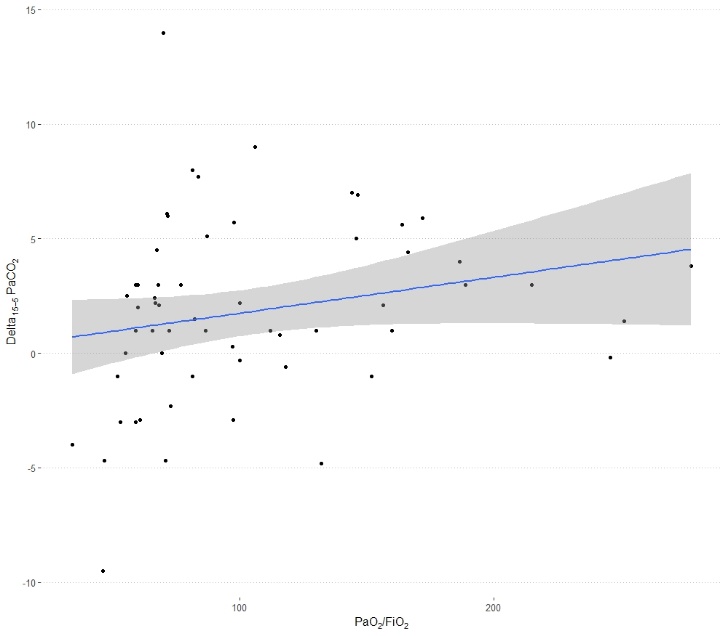 | 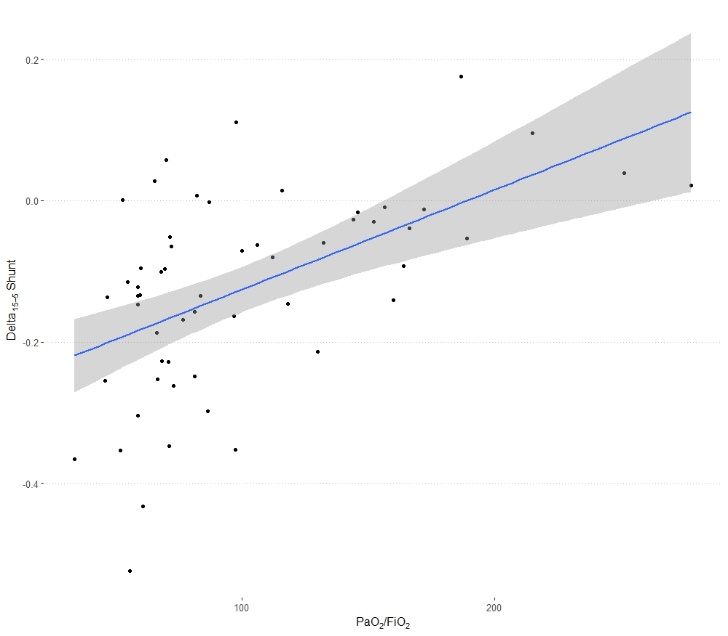 |
| 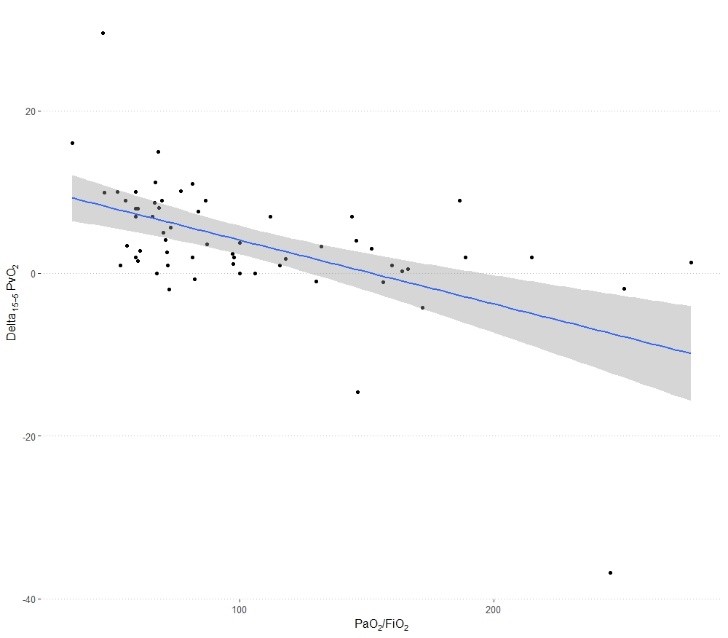 | 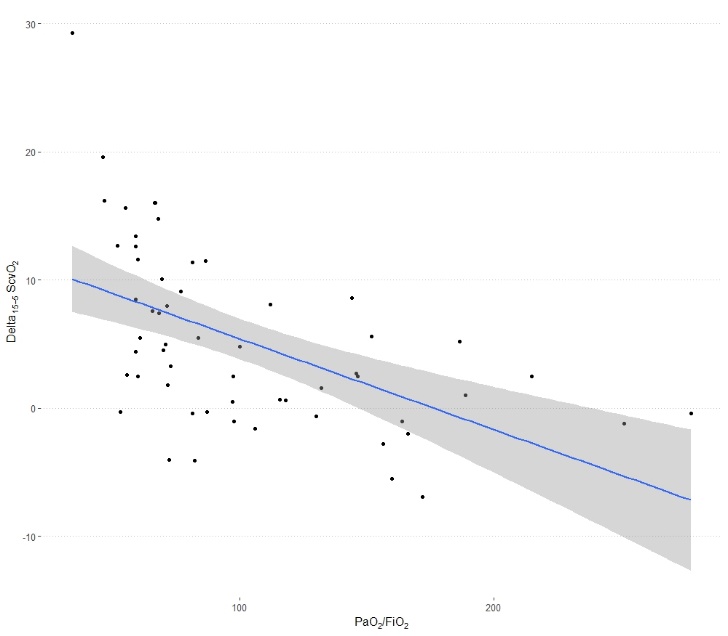 |
| 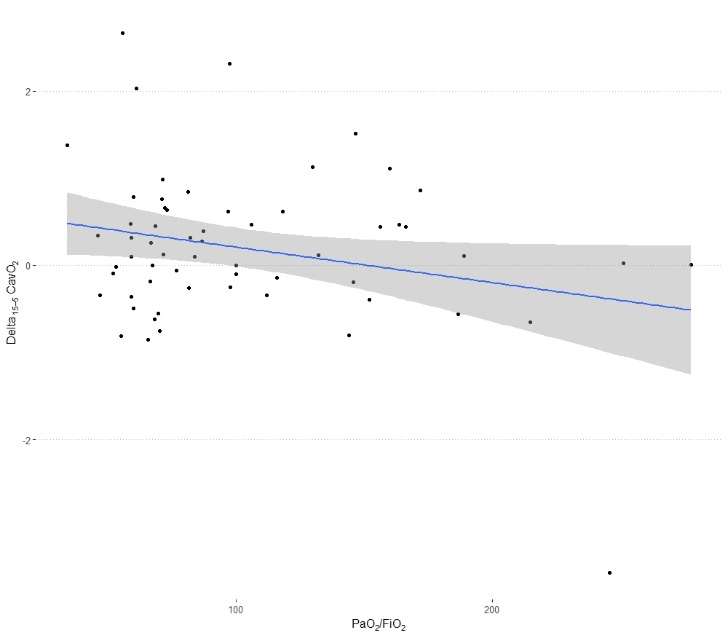 | 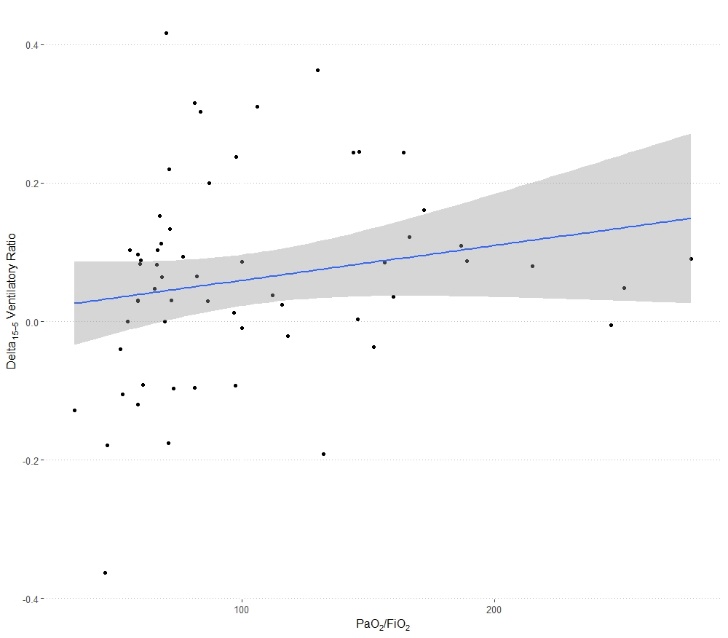 |
| 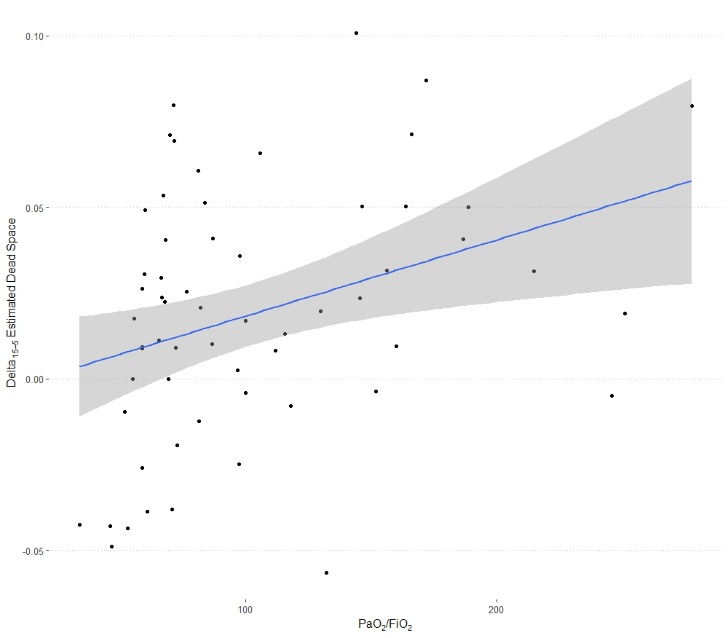 |  |

| Variables | R^2^ |
| --- | --- |
| Δ_15-5_ Plateau pressure, *cmH_2_O* | 0.011 |
| Δ_15-5_ Driving pressure, *cmH_2_O* | 0.100 |
| Δ_15-5_ Respiratory system elastance, *cmH_2_O/L* | 0.018 |
| Δ_15-5_ Lung elastance, *cmH_2_O/L* | 0.018 |
| Δ_15-5_ Chest wall elastance, *cmH_2_O/L* | 0.137 |
| Δ_15-5_ Lung stress, *cmH_2_O* | 0.003 |
| Δ_15-5_ Mechanical power, *J/min* | 0.008 |
| Δ_15-5_ PaO_2_, *mmHg* | 0.141 |
| Δ_15-5_ Right-to-left shunt, *%* | 0.272 |
| Δ_15-5_ PvO_2_, *mmHg* | 0.283 |
| Δ_15-5_ ScvO_2_, *%* | 0.276 |
| Δ_15-5_ CavO_2_, *mL* | 0.049 |
| Δ_15-5_ PaCO_2_, *mmHg* | 0.032 |
| Δ_15-5_ Ventilatory ratio | 0.021 |
| Δ_15-5_ Estimated physiological dead space | 0.097 |

**Table 4S.** Respiratory mechanics and gas exchange within 5, 15 cmH_2_O of PEEP and an empirical level of PEEP.

| **Variables** | **PEEP 5**  **29 patients** | **PEEP 15**  **29 patients** | **Empirical** **PEEP**  **29 patients** | ***p*** |
| --- | --- | --- | --- | --- |
| Plateau pressure, *cmH_2_O* | 17 ± 3 | 27 ± 2* | 32 ± 3*° | ***<0.001*** |
| Driving pressure, *cmH_2_O* | 11 [10 – 13] | 12 [10 – 13] | 13 [11 – 15]*° | ***<0.001*** |
| Respiratory system elastance, *cmH_2_O/L* | 24 [20 – 27] | 24 [22 – 29] | 28 [23 – 32]*° | ***<0.001*** |
| Lung elastance, *cmH_2_O/L* | 19 [16 – 23] | 19 [17 – 23] | 22 [17 – 27]° | ***0.049*** |
| Chest wall elastance, *cmH_2_O/L* | 4 [3 – 5] | 4 [3 – 6] | 5 [3 – 7] | *0.239* |
| Lung stress, *cmH_2_O* | 13.5 ± 3.2 | 21.5 ± 2.4* | 26.4 ± 2.8*° | ***<0.001*** |
| Mechanical power, *J/min* | 13.8 [11.2 – 15.8] | 22.3 [20.3 – 25.1]* | 26.8 [24.1 – 30.7]*° | ***<0.001*** |
| Mechanical Power_Compliance_rs_, *J/min/(mL/cmH_2_O)* | 0.34 ± 0.12 | 0.55 ± 0.14* | 0.75 ± 0.21*° | ***<0.001*** |
| PaO_2_, *mmHg* | 59 [54 – 69] | 81 [73 - 98]* | 84 [76 – 101]* | ***<0.001*** |
| PaO_2_/FiO_2_, *mmHg* | 81 [59 – 133] | 139 [85 – 182]* | 152 [111 – 202]*° | ***<0.001*** |
| Right-to-left shunt, *%* | 49 [41 – 54] | 38 [28 – 42]* | 32 [23 – 43]* | ***<0.001*** |
| PvO_2_, *mmHg* | 44 ± 7 | 49 ± 8* | 50 ± 6* | ***<0.001*** |
| ScvO_2_, *%* | 64 ± 10 | 78 ± 8^*^ | 76 ± 12 | ***0.023*** |
| C_a-v_O_2_, *mL* | 14.9 ± 2.2 | 16.0 ± 2.2* | 16.0 ± 2.1* | ***<0.001*** |
| PaCO_2_, *mmHg* | 50 [45 – 54] | 51 [47 – 55]* | 53 [50 – 55]* | ***0.026*** |
| Ventilatory ratio | 1.6 [1.4 – 1.9] | 1.6 [1.4 – 2.0] | 1.7 [1.5 – 2.1]* | ***0.026*** |
| Estimated physiological dead space | 0.47 ± 0.12 | 0.50 ± 0.09 | 0.62 ± 0.07*° | ***<0.001*** |

One-Way-ANOVA for repeated measures or Friedman test were performed, as appropriate; *: vs PEEP 5, °: vs PEEP 15. Tidal volume and respiratory rate were unchanged among the three PEEP levels. C_a-v_O_2_: arterial-venous oxygen content difference; Compliance_rs_: respiratory system compliance; ScvO_2_ central oxygen venous saturation; PvO_2_ mixed venous oxygen tension.

**Figure 7S.** Boxplot and three-point graphical representation showing each patient’s variable at 5, 15 cmH_2_O of PEEP and at Empirical PEEP.

| 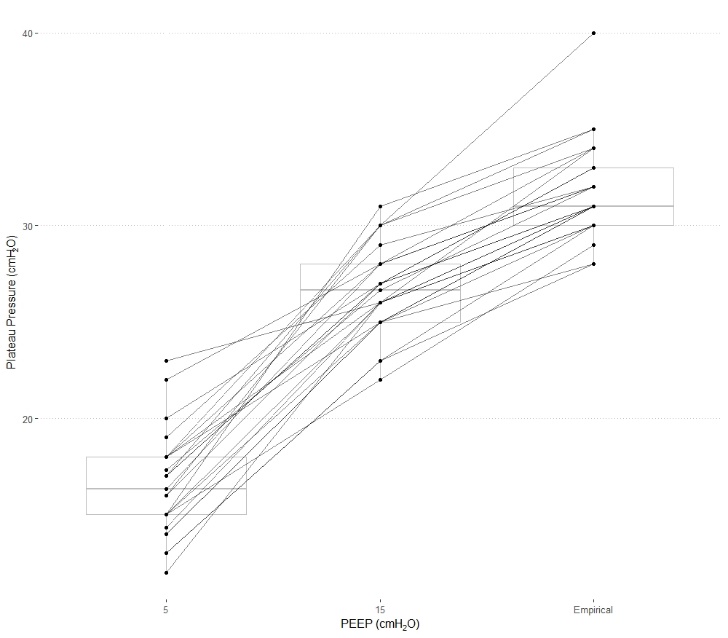 | 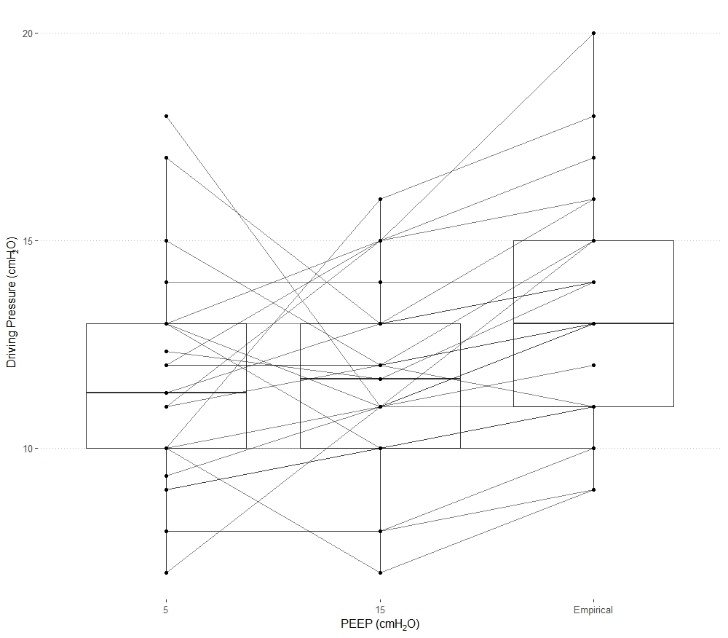 |
| --- | --- |
| 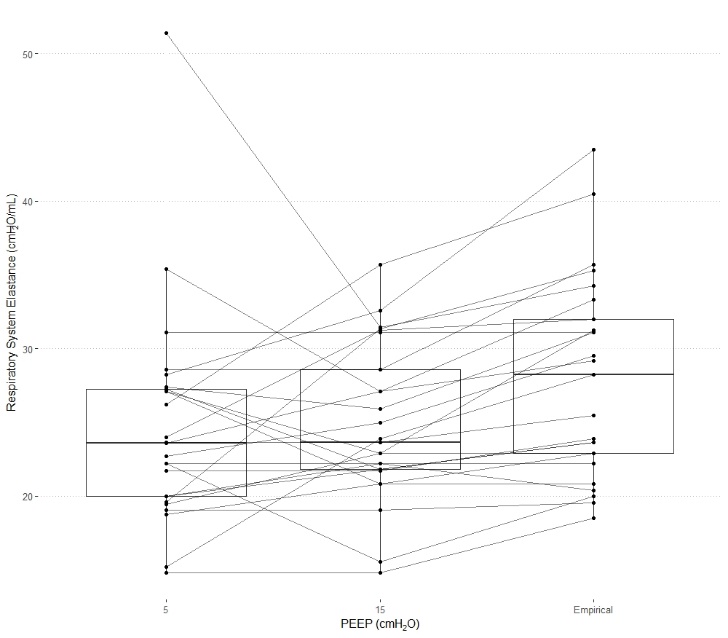 | 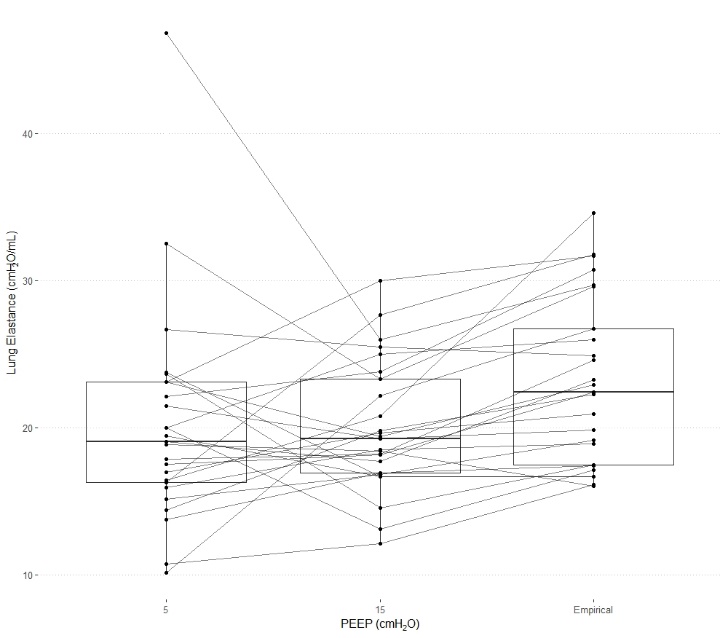 |
| 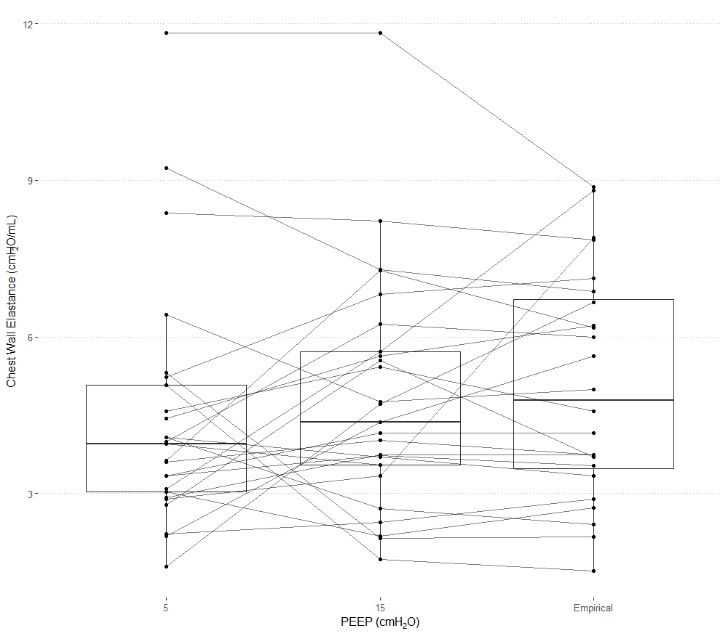 | 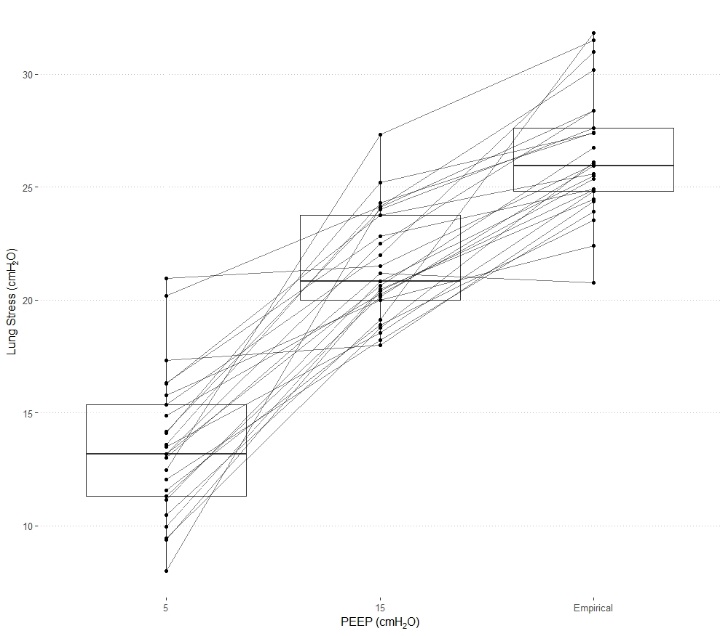 |
| 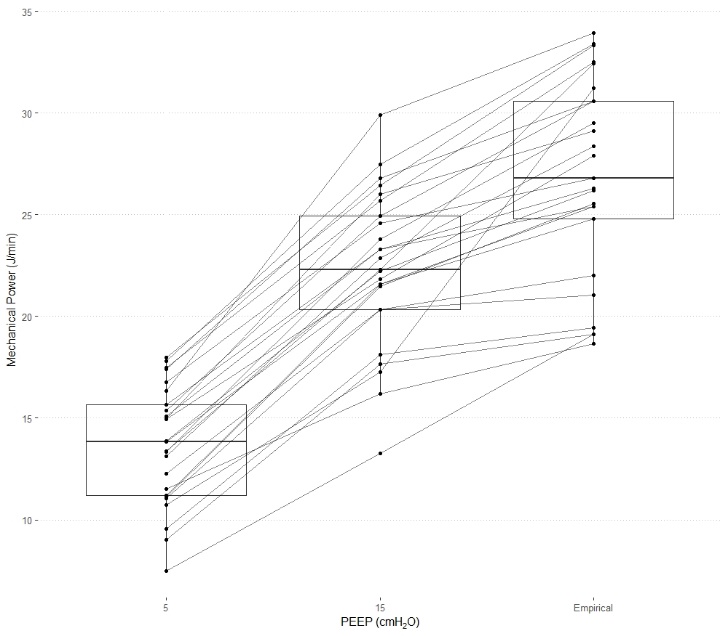 | 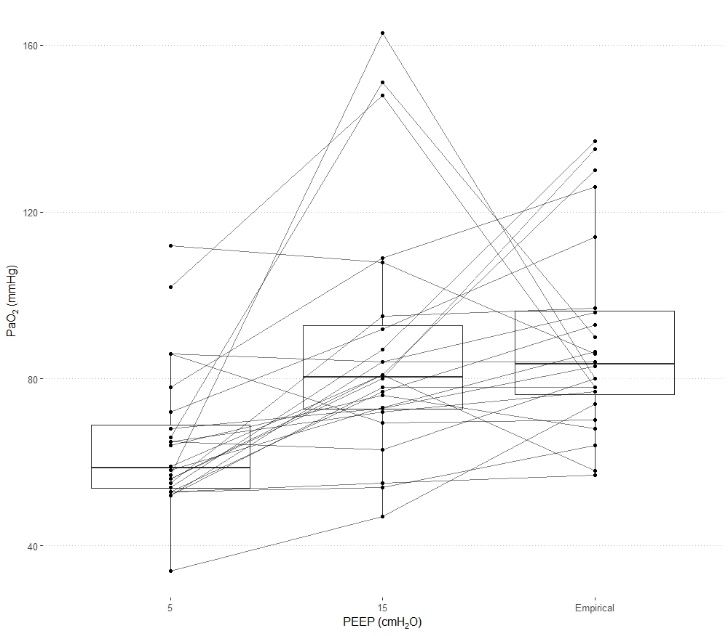 |
| 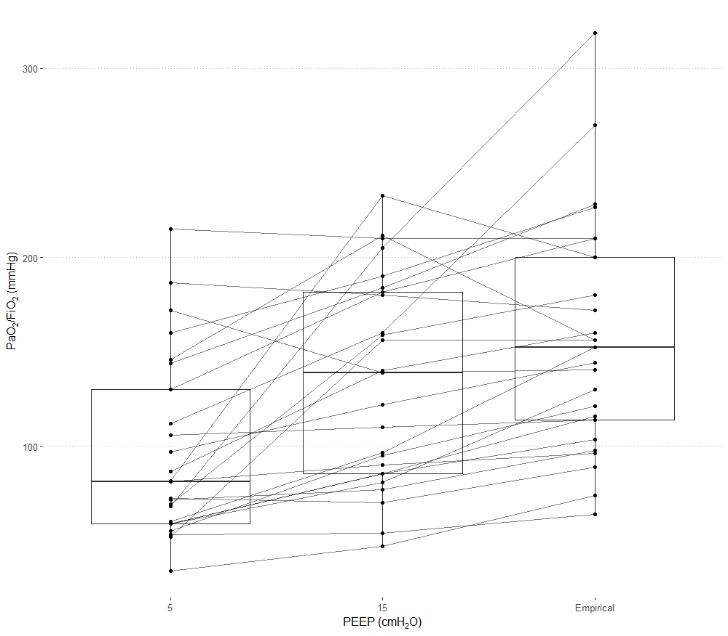 | 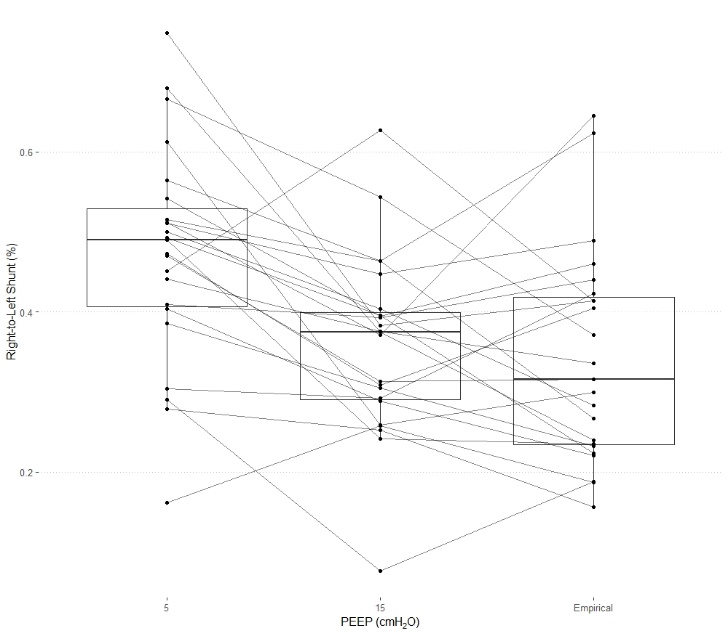 |
| 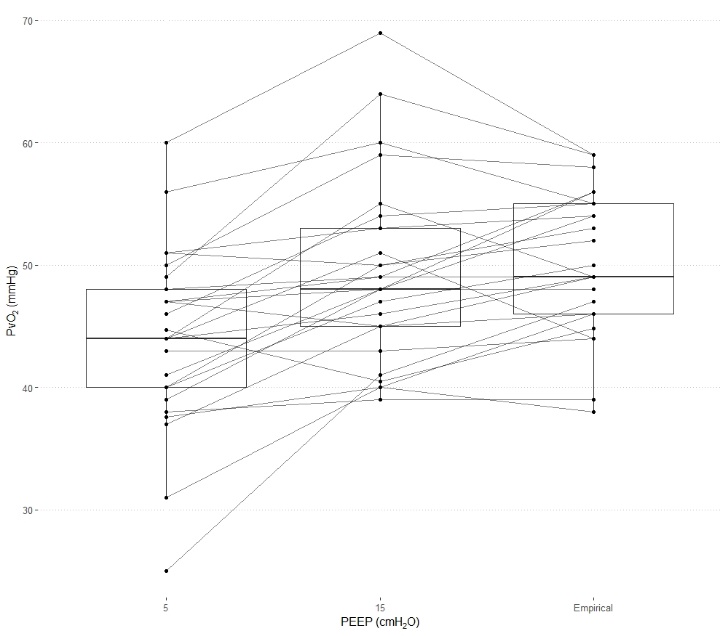 | 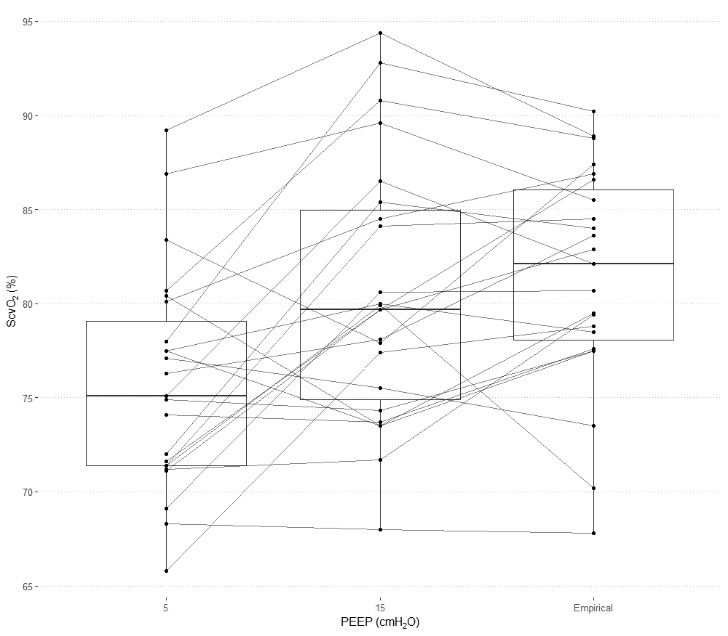 |
| 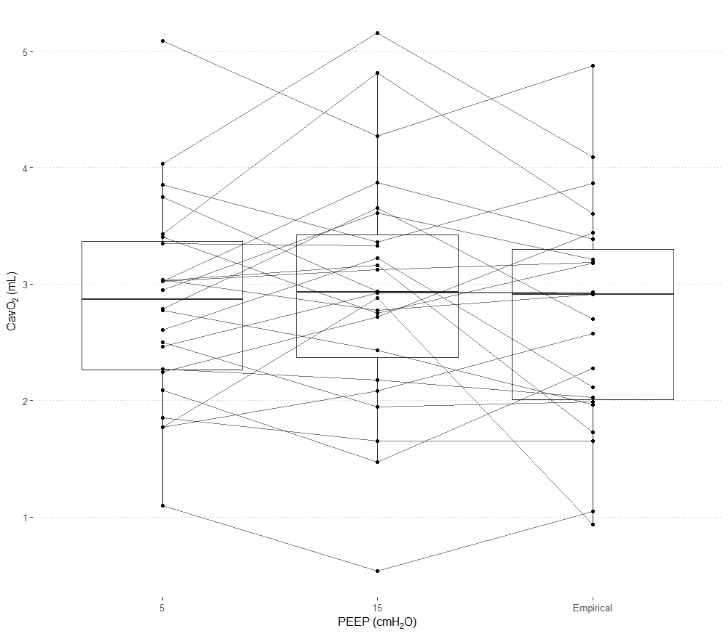 | 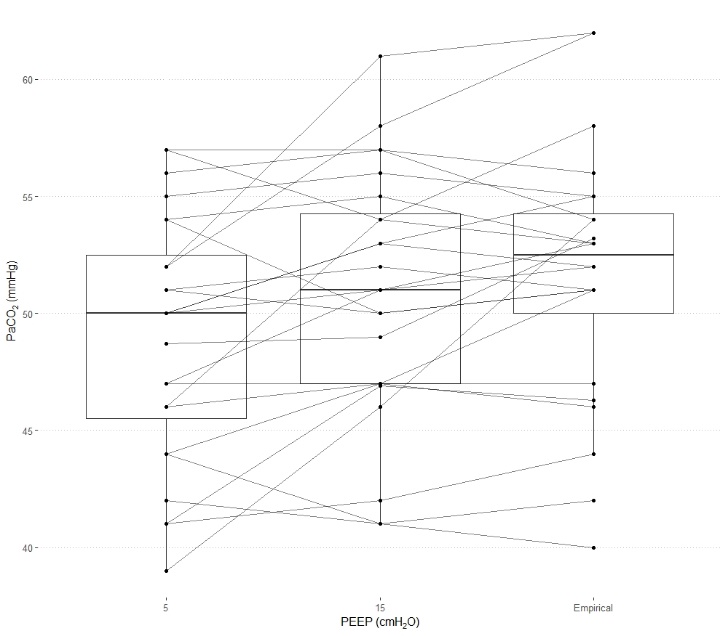 |
| 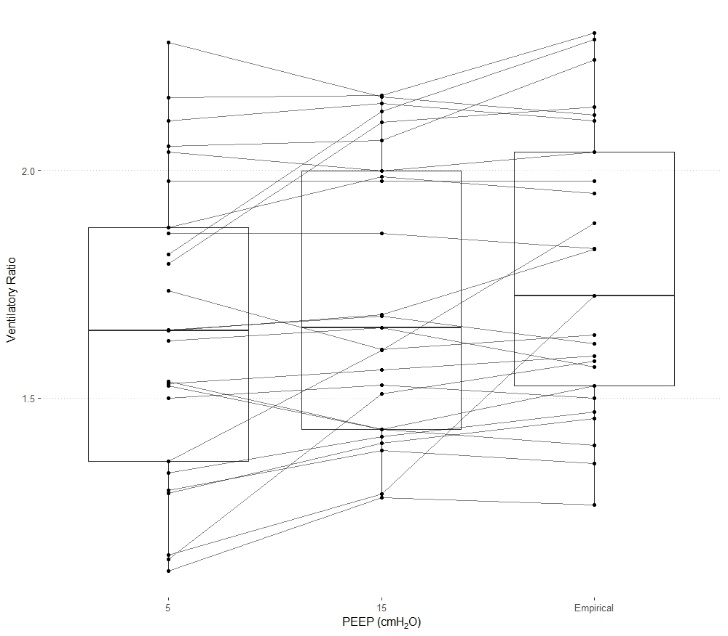 |  |
